# High salt activates the mineralocorticoid receptor-RUNX1 axis to mediate renal injury via mitochondrial dysfunction

**DOI:** 10.1186/s12967-026-08805-8

**Published:** 2026-08-11

**Authors:** Xi Zhang, Zhaode Feng, Hao Li, Teng Zhang, Mingfei Du, Ziyue Man, Zuyi Yuan, Jianjun Mu

**Affiliations:** 1 Department of Cardiovascular Medicine, First Affiliated Hospital of Xi’an Jiaotong University, 277 Yanta West Street, Xi’an, 710061 China; 2 Key Laboratory of Molecular Cardiology of Shaanxi Province, Xi’an, China; 3 International Joint Research Center for Cardiovascular Precision Medicine of Shaanxi Province, Xi’an, China; 4 Center for Mitochondrial Biology and Medicine, The Key Laboratory of Biomedical Information Engineering of Ministry of Education, School of Life Science, Xi’an Jiaotong University, Xi’an, China

**Keywords:** Mineralocorticoid receptor, RUNX1, Mitochondrial dysfunction, Salt-sensitive hypertension, Renal injury

## Abstract

**Background:**

Excessive salt intake contributes to salt-sensitive hypertension and renal injury in part through aberrant activation of the mineralocorticoid receptor (MR). While mitochondrial dysfunction is increasingly implicated in salt-induced target-organ damage, the transcriptional mechanisms linking MR overactivation to mitochondrial impairment remain unclear. By integrating cross-species transcriptomics with human genome-wide association study (GWAS) data, we sought to uncover potential pathogenic mediators contributing to salt-sensitive renal injury.

**Methods:**

Kidney transcriptomic data from Dahl salt-sensitive (DSS) rats, DOCA-salt rats, and human hypertensive nephropathy were integrated with human GWAS and GeneBridge renal transcriptomic resources to prioritize renal injury-associated mediators. Human renal tubular epithelial (HK-2) cells were then exposed to high-salt conditions to model tubular injury and functionally assess Runt-related transcription factor 1 (RUNX1) as a prioritized candidate mediator. Luciferase reporter assays and genetic manipulations were performed to elucidate the transcriptional mechanisms coupling MR activation to mitochondrial dysfunction. Pharmacological interventions with the MR antagonist finerenone and the RUNX1 inhibitor Ro5-3335 were further conducted in high-salt-fed DSS rats to evaluate the translational potential of this pathogenic cascade.

**Results:**

Integrative genomic analysis identified RUNX1 as a conserved candidate mediator markedly upregulated in salt-sensitive renal injury. Mechanistically, high-salt stimulation promoted MR-dependent RUNX1 transcriptional activation. Aberrant RUNX1 upregulation contributed to mitochondrial damage and cytosolic mitochondrial DNA (mtDNA) release, which in turn engaged the cGAS-STING innate immune pathway and promoted renal tubular inflammatory responses and tubulointerstitial fibrosis. Genetic knockdown of RUNX1 attenuated these mitochondrial and inflammatory alterations, whereas RUNX1 overexpression exacerbated them. The MR antagonist finerenone exerted significant renoprotective effects by suppressing MR-RUNX1 signaling and downstream mitochondrial and inflammatory injury. Furthermore, in vivo pharmacological inhibition of RUNX1 with Ro5-3335 effectively attenuated mitochondrial dysfunction and renal pathological damage, supporting RUNX1 as a therapeutically relevant mediator.

**Conclusions:**

Our study identifies RUNX1 as an important transcriptional mediator linking aberrant MR signaling to mitochondrial dysfunction, inflammatory activation, and fibrotic remodeling. Targeting the MR-RUNX1 axis represents a promising therapeutic strategy for mitigating salt-sensitive hypertension and renal injury.

**Graphical abstract:**

**Figure:**
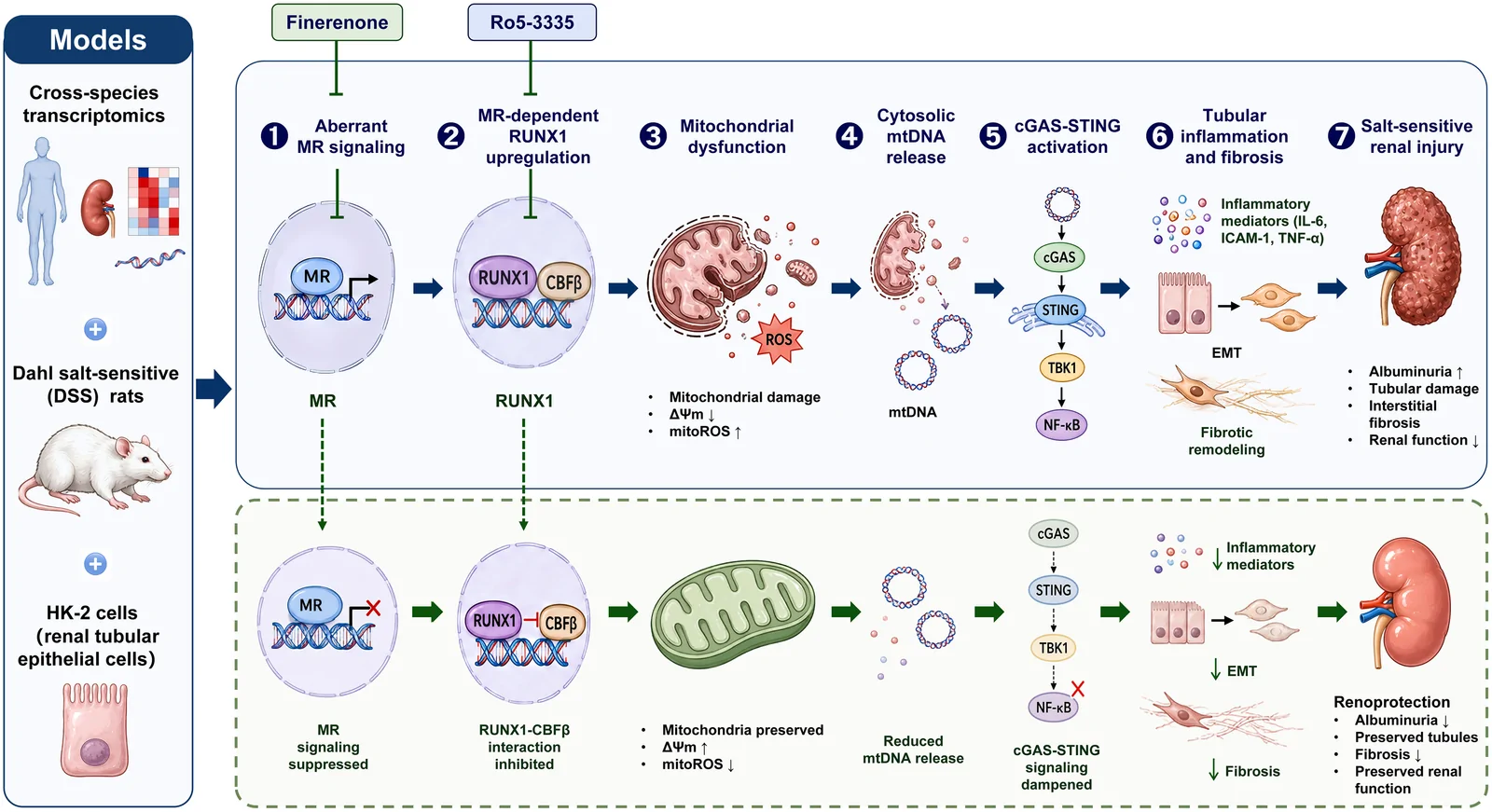

**Supplementary Information:**

The online version contains supplementary material available at https://doi.org/10.1186/s12967-026-08805-8.

## Introduction

Hypertension represents a global public health crisis, claiming over 10 million lives annually through cardiovascular events and chronic kidney disease (CKD) [1, 2]. Among its diverse etiologies, excessive salt intake contributes substantially to both blood pressure elevation and target-organ damage [3]. An exaggerated blood pressure response to salt load defines salt sensitivity, a pathophysiological trait intimately linked to increased cardiovascular and renal morbidity [4]. Mechanistically, the mineralocorticoid receptor (MR) emerges as an important mediator of this specific renal injury. Under high-salt conditions, the MR undergoes aberrant, aldosterone-independent activation [5], triggering oxidative stress and inflammatory cascades that precipitate hypertensive nephropathy [6]. While finerenone, a highly selective non-steroidal MR antagonist, has demonstrated remarkable efficacy in mitigating diabetic kidney disease [7, 8], its renoprotective potential and downstream molecular mechanisms in salt-sensitive renal injury remain largely elusive.

As a high-energy-demand organ, the kidney relies heavily on mitochondrial adenosine triphosphate (ATP) to power tubular sodium transport [9]. Emerging evidence indicates that salt overload compromises mitochondrial structural integrity and bioenergetics [10]. At the ultrastructural level, this deterioration manifests as profound mitochondrial swelling, cristae disorganization, and fragmentation [11, 12]. Importantly, mitochondrial dysfunction is not merely a metabolic deficit but an immunogenic trigger. The aberrant release of mitochondrial DNA (mtDNA) into the cytosol serves as a potent damage-associated molecular pattern (DAMP) that engages the cyclic GMP-AMP synthase (cGAS)-stimulator of interferon genes (STING) pathway, driving inflammatory cell recruitment and progressive fibrosis [13]. Despite evidence that MR inhibition restores mitochondrial homeostasis in diabetic models [14], the specific intracellular effectors translating MR overactivation into mitochondrial-driven innate immunity during salt overload remain poorly defined.

Runt-related transcription factor 1 (RUNX1), an evolutionarily conserved master regulator, is classically recognized for its roles in hematopoiesis [15, 16]. However, recent transcriptomic insights have highlighted its emerging pathogenic roles in renal diseases. For instance, RUNX1 has been implicated in sepsis-induced acute kidney injury (AKI) and has been reported to promote epithelial-mesenchymal transition (EMT) and fibrosis via TGF-β signaling [17, 18]. Furthermore, RUNX1 has been shown to interact with NF-κB p50 and amplify inflammatory responses [19]. Importantly, RUNX1 is pharmacologically targetable; the small-molecule RUNX1 inhibitor Ro5-3335 has demonstrated protective effects in experimental models of AKI and cardiovascular disorders [20, 21]. Guided by our integrative screening strategy, we identified a strong association between RUNX1, mitochondrial dysfunction, and salt-sensitive renal injury. Consequently, we hypothesized that aberrant MR activation contributes to renal injury by promoting RUNX1 upregulation and mitochondrial dysfunction, and that targeted suppression of this transcription factor could offer therapeutic benefit in this pathological context.

To test this hypothesis, we integrated cross-species kidney transcriptomic profiling, human genome-wide association study (GWAS) evidence, and public kidney expression resources, followed by comprehensive functional investigations in vivo and in vitro. Here, we show that high salt promotes MR-dependent RUNX1 transcriptional activation, which contributes to mitochondrial dysfunction, cytosolic mtDNA release, and engagement of the cGAS-STING innate immune pathway. Furthermore, pharmacological targeting of the MR-RUNX1 cascade with finerenone or Ro5-3335 effectively ameliorated mitochondrial abnormalities and renal injury in high-salt-fed Dahl salt-sensitive (DSS) rats. Together, our findings suggest an MR-RUNX1 signaling mechanism that links high-salt exposure to mitochondrial dysfunction, renal tubular inflammation, and fibrotic remodeling, highlighting RUNX1 as a therapeutically relevant mediator of salt-sensitive renal injury.

## Methods

### Animal studies

A total of 48 male Dahl salt-sensitive (DSS) rats aged 8 weeks were obtained from Vital River Laboratory Animal Technology Co., Ltd. (Beijing, China) and used in this study. All animals were housed in standard cages under specific pathogen-free (SPF) conditions at the Animal Center of Xi’an Jiaotong University Health Science Center, with three rats per cage, and were maintained at 22 ± 1 °C with a 12-hour light/dark cycle and ad libitum access to food and water. After one week of acclimatization, the rats were randomly assigned to the experimental groups (*n* = 6 per group) for an 8-week regimen.

To investigate the dose-dependent renoprotective effects of finerenone, the first cohort of rats was divided into five groups: (i) normal-salt (NS) group: fed a 0.3% NaCl diet; (ii) high-salt (HS) group: fed an 8% NaCl diet; and (iii-v) finerenone-treated groups (HS + Fin): fed an 8% NaCl diet and treated with finerenone via oral gavage once daily at doses of 5, 10, or 20 mg/kg/day. Finerenone was supplied as powder and purchased from MedChemExpress (MCE, Monmouth Junction, NJ, USA; Cat. No. HY-111372) and was dissolved in 10% ethanol, 40% polyethylene glycol 400 (PEG400), and 50% water before administration.

To investigate the downstream pathological role of RUNX1, an independent cohort of rats was assigned to three groups: (i) NS vehicle group: fed a 0.3% NaCl diet and received subcutaneous vehicle injections; (ii) HS vehicle group: fed an 8% NaCl diet and received subcutaneous vehicle injections; and (iii) Ro5-3335-treated (HS+Ro5) group: fed an 8% NaCl diet and treated with the RUNX1 inhibitor Ro5-3335 via subcutaneous injection every other day at a dose of 10 mg/kg. Ro5-3335 was supplied as powder and purchased from MedChemExpress (MCE; Cat. No. HY-108470) and was dissolved in 10% dimethyl sulfoxide (DMSO), 40% PEG400, 5% Tween-80, and 45% saline before administration.

At the end of the intervention, rats were anesthetized with 2% pentobarbital sodium and euthanized for tissue collection. Animal carcasses and biological remnants were transferred to the designated collection point of the institutional animal facility and subsequently handled through centralized biosafety-compliant disposal in accordance with institutional regulations. All animal procedures were approved by the Ethics Committee of Xi’an Jiaotong University (No. XJTUAE2025-3809) and conducted in strict accordance with the Guide for the Care and Use of Laboratory Animals.

### Cell culture

Human renal tubular epithelial (HK-2) cells were purchased from Wuhan Servicebio Technology Co., Ltd. (Wuhan, China). Cells were cultured in Dulbecco’s Modified Eagle Medium/Nutrient Mixture F-12 (DMEM/F-12; Gibco) supplemented with 10% fetal bovine serum (FBS) and 1% penicillin-streptomycin in a humidified incubator at 37 °C with 5% CO₂. Upon reaching approximately 60–70% confluence, cells were synchronized by serum starvation for 12 h before treatments. The experimental groups were defined as follows: (i) Control group: maintained in basal medium; (ii) Osmotic control group: basal medium supplemented with 40 mM mannitol; (iii) High-salt group: basal medium supplemented with an additional 40 mM NaCl; (iv) Finerenone treatment groups: HS medium supplemented with finerenone at varying concentrations (0.2, 0.4, 0.8, 1, 2, 5, and 10 µM) for 48 h. Finerenone was dissolved in DMSO, with the final DMSO concentration kept below 0.1% in all groups to exclude vehicle effects.

### Lentiviral vector construction and transduction

To modulate RUNX1 expression, lentiviral vectors were designed and packaged by WZ Biosciences (Jinan, China). For gene knockdown, short hairpin RNAs (shRNAs) targeting human RUNX1 were cloned into the pLent-U6-shRNA-CMV-GFP-P2A-Puro vector. For gene overexpression, the full-length human RUNX1 coding sequence was cloned into the pLent-EF1a-FH-CMV-GFP-P2A-Puro vector. Corresponding empty vectors (shNC and oeNC) served as negative controls. For transduction, HK-2 cells were seeded in 12-well plates. Upon reaching 50–60% confluence, cells were infected with lentiviral particles at an optimal multiplicity of infection (MOI) of 20 in the presence of 5 µg/mL ADV-HR to maximize efficiency. After 24 h of infection, the medium was replaced with fresh complete medium. Stable cell lines were established by selection with 2 µg/mL puromycin for 5 days. Transduction efficiency was visualized via green fluorescent protein (GFP) fluorescence, and the knockdown or overexpression of RUNX1 was rigorously validated by Western blot analysis prior to subsequent experiments.

### Blood pressure measurements

Systolic blood pressure (SBP) was monitored weekly in conscious rats using a non-invasive tail-cuff plethysmography system (BP-2000; Visitech Systems, Apex, NC, USA), strictly adhering to standardized protocols [22, 23]. To minimize stress-induced fluctuations, all rats underwent a one-week acclimatization training involving restraint and cuff inflation prior to the formal study. During measurement sessions, rats were placed on a warming platform maintained at 37 °C to ensure adequate caudal artery dilation and pulse detection. Measurements were conducted at a fixed time of day to avoid circadian variations. In each session, 10 preliminary measurements were performed to stabilize the animals, followed by 10 actual measurements recorded at 2-minute intervals. The final SBP value was derived from the arithmetic mean of these 10 actual readings.

### Sample collection and biochemical analysis

To assess renal function, 24-hour urine samples were collected using individual metabolic cages. Urine volume was recorded, and samples were centrifuged at 3,000 × g for 10 min at 4 °C to remove debris before storage at -80 °C. At the end of the experiment, terminal blood samples were obtained via abdominal aorta puncture under anesthesia. Serum was isolated by centrifugation at 1,500 × g for 10 min at 4 °C and aliquoted for analysis.

Key renal functional parameters, including blood urea nitrogen (BUN), serum creatinine (SCr), 24-h urinary albumin (uALB), and urinary creatinine (uCRE), were quantified using an automatic biochemical analyzer (Hitachi High-Technologies, Tokyo, Japan). The urinary albumin-to-creatinine ratio (UACR), a sensitive marker of glomerular filtration barrier injury, was calculated using the formula: UACR (mg/µmol) = uALB (mg/L) / uCRE (µmol/L). For the Ro5-3335 cohort, endpoint serum biochemical parameters, including alanine aminotransferase (ALT), aspartate aminotransferase (AST), albumin (ALB), total protein (TP), and lactate dehydrogenase (LDH), were also measured using the automatic biochemical analyzer to provide a limited serum biochemical safety assessment.

### Histological and immunohistochemical analyses

Renal tissues were fixed in 4% paraformaldehyde, embedded in paraffin, and sectioned at 4 μm thickness. Sections were stained with hematoxylin and eosin (H&E), Masson’s trichrome, and Sirius Red to evaluate renal injury and fibrosis. For immunohistochemistry (IHC), sections were incubated overnight at 4 °C with specific primary antibodies against RUNX1 and NF-κB p65 (Table S1). Subsequently, sections were treated with horseradish peroxidase (HRP)-conjugated secondary antibodies for 1 h at room temperature. Immunoreactivity was visualized using diaminobenzidine (DAB), followed by hematoxylin counterstaining. Images were captured using a light microscope, and positive staining was quantified using ImageJ software.

### Transmission electron microscopy (TEM)

Mitochondrial ultrastructure was examined by standard TEM procedures. Fresh renal cortical tissues (1 mm^3^) and HK-2 cells were fixed overnight at 4℃ in a solution containing 2% paraformaldehyde and 2.5% glutaraldehyde. Samples were post-fixed with 1% osmium tetroxide, dehydrated in graded ethanol, and embedded in epoxy resin. Ultrathin Sect.  (70 nm) were stained with uranyl acetate and lead citrate and analyzed using a JEOL transmission electron microscope (JEOL Ltd., Tokyo, Japan).

### Western blotting

Total protein was extracted from renal tissues and HK-2 cells using radioimmunoprecipitation assay (RIPA) lysis buffer supplemented with protease and phosphatase inhibitor cocktails. Protein concentration was determined using a bicinchoninic acid (BCA) Protein Assay Kit (Thermo Fisher Scientific, Waltham, MA, USA). Equal amounts of protein were resolved by sodium dodecyl sulfate-polyacrylamide gel electrophoresis (SDS-PAGE) and electrotransferred onto polyvinylidene difluoride (PVDF) membranes. Following blocking, membranes were incubated with specific primary antibodies (Table S1), followed by appropriate HRP-conjugated secondary antibodies. Immunoreactive bands were visualized using an enhanced chemiluminescence (ECL) detection system and quantified via ImageJ software. Relative protein expression levels were normalized to GAPDH.

### RNA extraction and quantitative real-time PCR (qPCR)

Total RNA was isolated from renal tissues and HK-2 cells using TRIzol reagent (Thermo Fisher Scientific). RNA concentration and purity were assessed using a spectrophotometer. Subsequently, total RNA was reverse-transcribed into complementary DNA (cDNA) using the Evo-MLV Reverse Transcription Kit (Accurate Biology, Changsha, China). Real-time PCR amplification was performed on a Bio-Rad iQ5 Real-Time PCR System (Bio-Rad, Hercules, CA, USA) utilizing the SYBR Green Pro Taq HS Premix (Accurate Biology). Specific primer sequences are listed in Table S2. Each qPCR reaction was performed in technical triplicate. Relative mRNA expression levels were normalized to the endogenous control GAPDH and calculated using the 2^−ΔΔCt^ method.

### RNA sequencing (RNA-seq) and Data Processing

Total RNA was extracted from renal cortical tissues of DSS rats (NS group, *n* = 6; HS group, *n* = 5) and cultured HK-2 cells (shNC + HS and shRUNX1 + HS groups; *n* = 6 per group) using TRIzol reagent. RNA integrity was rigorously assessed using an Agilent 2100 Bioanalyzer (Agilent Technologies, Santa Clara, CA, USA), and RNA concentration was quantified using NanoDrop spectrophotometry (Thermo Fisher Scientific). Only high-quality RNA samples meeting the criteria of RNA Integrity Number (RIN) ≥ 7.0 and total RNA amount ≥ 2 µg were used for library construction. Sequencing libraries were generated using the NEBNext Ultra II RNA Library Prep Kit (New England Biolabs, Ipswich, MA, USA) and sequenced on an Illumina NovaSeq X Plus platform (Illumina, San Diego, CA, USA) to generate 150-bp paired-end reads.

Raw paired-end RNA-seq reads were processed using fastp v0.22.0 for quality control and read trimming. Clean reads from HK-2 cells were aligned to the human GRCh38 reference genome with GENCODE release 45 basic gene annotation using Spliced Transcripts Alignment to a Reference (STAR) v2.7.10b, whereas clean reads from rat kidney tissues were aligned to the mRatBN7.2 reference genome with Ensembl release 113 gene annotation using STAR v2.7.10b. Coordinate-sorted Binary Alignment Map (BAM) files were generated for subsequent quantification. Gene-level read counts were quantified using featureCounts v2.0.6 in paired-end mode, with exonic reads summarized by gene_id. The resulting raw gene count matrices were used for downstream differential expression analysis.

### Differential expression analysis of RNA-seq data

For differential expression analysis, lowly expressed genes were filtered, retaining only those with more than 5 reads in at least half of the examined samples. RNA-seq count data were normalized by the trimmed mean of M-values (TMM) method via the calcNormFactors function in edgeR (v3.34.1) [24]. Differential expression analysis was performed by linear modeling implemented in limma (v3.48.3) [25], with design matrices constructed according to the experimental groups for each comparison. *P*-values were adjusted for multiple testing using the Benjamini–Hochberg false discovery rate (FDR) method, and genes with adjusted *P* < 0.05 and |log2 fold change| > 1.5 were considered significantly differentially expressed.

### Gene set enrichment analysis

Enrichment analysis of differentially expressed genes (DEGs) in each comparison was performed with the fgsea package (v1.30.0) [26]. All genes were preranked according to their log₂ fold change values, and enrichment was assessed using gene sets from the Gene Ontology (GO), Kyoto Encyclopedia of Genes and Genomes (KEGG), and Reactome pathway databases. Significance was determined based on adjusted p-values (FDR < 0.05) derived from 10,000 permutations.

### Genome-wide association analysis (GWAS)

Summary statistics for blood mitochondrial DNA copy number (mtDNA-CN) GWAS were obtained from the GWAS catalog (GCST90268497) [27]. Manhattan plots were generated with the CMplot package (v4.5.1) in R, displaying −log10(p-value) against genomic position across all autosomes, with genome-wide (*P* = 5 × 10^− 8^) and suggestive (*P* = 1 × 10^− 6^) significance thresholds indicated. Variants within the RUNX1 locus were further examined using regional association plots, in which linkage disequilibrium (LD) patterns were determined based on the European population from the 1000 Genomes Project (phase 3) and visualized with locuszoomr (v0.3.4).

### Phenotype association analysis

Associations between RUNX1 and human phenotypes were evaluated using the Human Genetics Amplifier (HuGeAMP) platform (https://hugeamp.org/gene.html?gene=RUNX1), which integrates GWAS summary statistics from multiple disease- and trait-specific knowledge portals [28].

### Gene-module association analysis

Gene–pathway associations were evaluated by the Gene–Module Association Determination (G-MAD) analysis implemented in the GeneBridge (https://www.systems-genetics.org/genebridge) [29]. This analysis integrates large-scale renal transcriptomic resources, comprising 30 human kidney datasets, each with more than 80 samples, to quantify the association strengths between individual genes and curated functional modules. For each dataset, gene expression profiles were correlated with module eigengenes derived from GO, KEGG, and Reactome pathway definitions. Gene–module association scores (GMAS) were calculated and meta-analyzed across datasets, with GMAS > 0.268 indicating a significant positive association and GMAS < − 0.268 indicating a significant negative association.

### Dual-luciferase reporter assay

To assess MR-dependent regulation of RUNX1 promoter activity, the human RUNX1 promoter region was amplified and cloned into the pGL3-Basic luciferase reporter vector. The cloning was performed using MluI and XhoI restriction sites and T4 DNA Ligase. Site-directed mutagenesis was performed using DpnI to disrupt the predicted MR binding motifs, generating mutant (MT) constructs: (i) MT-RUNX1-1 (mutation at Site 1); (ii) MT-RUNX1-2 (mutation at Site 2); (iii) MT-RUNX1-3 (combined mutations). All constructs were rigorously verified by Sanger sequencing (GENEWIZ).

HK-2 cells were seeded in 24-well plates and cultured to 60–70% confluence. Cells were co-transfected with 0.5 µg of wild-type (WT) or MT reporter plasmids and 0.05 µg of the internal control plasmid (pRL-TK) using Lipofectamine™ 2000 (Invitrogen) in Opti-MEM reduced-serum medium. Four hours post-transfection, the medium was replaced with complete DMEM/F12 for the following treatments: Control, HS (40 mM NaCl), and HS + Fin (40 mM NaCl + 1 µM finerenone). After 48 h, cells were lysed, and luciferase activities were measured using the Dual-Luciferase Reporter Assay System (Promega) on a GloMax bioluminescence detector (Promega). The relative luciferase activity was calculated as the ratio of Firefly to Renilla luminescence units (F/R). All assays were performed in triplicate.

### Immunofluorescence

HK-2 cells seeded on coverslips were subjected to the indicated treatments. To visualize mitochondria, cells were stained with MitoTracker Red CMXRos (Thermo Fisher Scientific) according to the manufacturer’s protocol prior to fixation. Subsequently, cells were fixed with 4% paraformaldehyde for 10 min, permeabilized with 0.25% Triton X-100 for 30 min, and blocked with 2% BSA for 1.5 h at room temperature. The slides were then incubated overnight at 4 °C with specific primary antibodies: anti-double-stranded DNA (dsDNA) (1:50; sc-58749, Santa Cruz) and anti-cGAS (1:50; A8335, ABclonal). Following phosphate-buffered saline (PBS) washes, cells were incubated with appropriate fluorophore-conjugated secondary antibodies for 1.5 h at room temperature in the dark. Nuclei were counterstained with 4′,6-diamidino-2-phenylindole (DAPI) (1 µg/mL). Fluorescence images were acquired using a Leica confocal laser-scanning microscope equipped with a 63× oil immersion objective.

### Mitochondrial reactive oxygen species (mitoROS) detection

Following pharmacological interventions, HK-2 cells were incubated with 2 µM MitoSOX™ Red reagent (Thermo Fisher Scientific) for 30 min at 37 °C in the dark. After three washes with Hank’s balanced salt solution (HBSS), mitochondrial superoxide production was visualized using a Leica confocal laser-scanning microscope.

### Mitochondrial membrane potential assessment

The mitochondrial transmembrane potential (ΔΨm) was evaluated using the JC-1 Assay Kit (Beyotime, Shanghai, China). Cells were incubated with JC-1 working solution for 20 min at 37 °C per the manufacturer’s protocol. Fluorescence signals were detected via confocal microscopy. Red fluorescence (JC-1 aggregates) indicated intact ΔΨm, while green fluorescence (JC-1 monomers) indicated mitochondrial depolarization.

### DNA isolation and mtDNA copy number quantification

Total genomic DNA was extracted from renal tissues and cells using the TIANamp Genomic DNA Kit (TIANGEN Biotech, Beijing, China). The relative mtDNA copy number was determined by qPCR [30, 31]. 50 ng of template DNA was amplified using specific primers targeting the mitochondrial gene MT-ND1 and the nuclear reference gene 18 S ribosomal RNA (18 S rRNA); primer sequences are listed in Table S2. The relative mtDNA copy number was calculated using the 2^−ΔΔCt^ method.

#### Quantification of cytosolic mtDNA

Cytosolic mtDNA was isolated and quantified following established protocols [32, 33]. HK-2 cells were divided into two equal aliquots. One aliquot was resuspended in PBS for total DNA extraction to serve as the normalization control. The other aliquot was resuspended in digitonin permeabilization buffer containing 25 µg/mL digitonin to selectively release cytosolic contents. The lysate was centrifuged at 1,000 × g to pellet intact cells and organelles. The supernatant was further centrifuged at 17,000 × g to yield the organelle-free cytosolic fraction. DNA was extracted from both fractions using the DNeasy Blood & Tissue Kit (TIANGEN Biotech). Cytosolic mtDNA levels were quantified by qPCR using mitochondrial-specific primers (MT-ND1, MT-RNR2, MT-CYB; sequences in Table S2) and normalized to the total mtDNA content. The purity of the cytosolic fractions was validated by Western blotting using GAPDH as a cytosolic marker, Lamin B1 as a nuclear marker, and cytochrome c oxidase subunit IV (COX IV) as a mitochondrial marker, to exclude detectable nuclear leakage or mitochondrial contamination during digitonin-based fractionation.

### Statistical Analysis

All data were analyzed using GraphPad Prism 9.5. Intergroup differences were assessed by the Student’s t-test, one-way analysis of variance (ANOVA), or two-way ANOVA followed by Tukey’s post hoc test for pairwise comparisons, as appropriate. Spearman correlation analysis was performed to determine relationships between variables. Statistical significance was defined as *P* < 0.05. Specific statistical tests applied to each dataset are detailed in the corresponding figure legends.

## Results

### Finerenone ameliorates salt-sensitive hypertension and renal injury

**Fig. 1:**
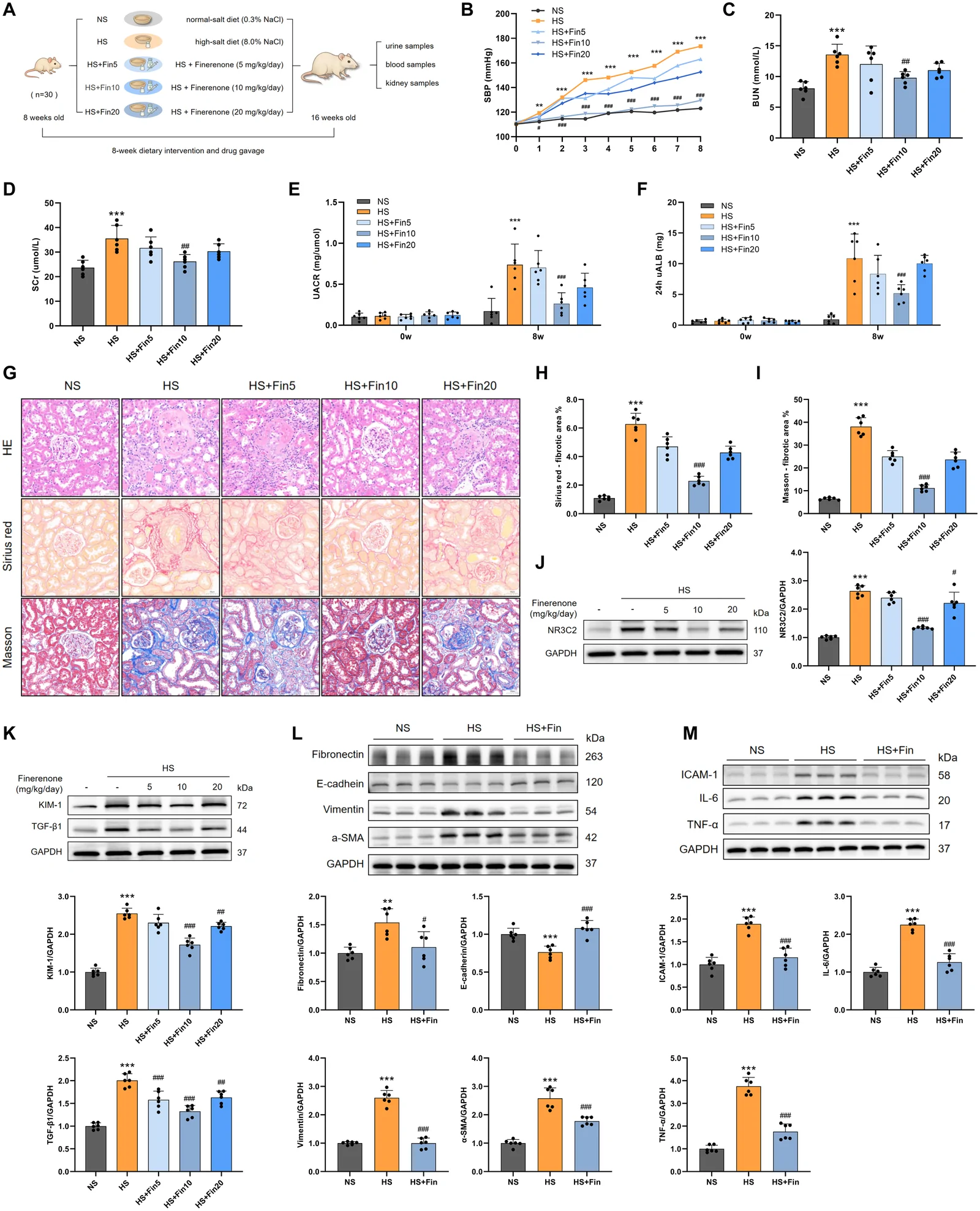
Finerenone ameliorates salt-sensitive hypertension and renal injury. (**A**) Schematic illustration of the experimental design. DSS rats were maintained on a NS or HS diet for 8 weeks with concomitant finerenone administration via gavage (5, 10, or 20 mg/kg/day). (**B**) Longitudinal monitoring of SBP changes throughout the 8-week intervention period. (**C**) Quantification of BUN levels. (**D**) Quantification of SCr levels. (**E**) Measurement of UACR. (**F**) Measurement of 24-hour uALB excretion. (**G**) Representative photomicrographs of DSS rat kidney sections stained with H&E, Sirius Red, and Masson’s trichrome (*n* = 6 rats per group; scale bar = 50 μm). (**H**) Quantitative analysis of the tubulointerstitial fibrotic area assessed by Sirius Red staining. (**I**) Quantitative analysis of the collagen deposition area assessed by Masson’s trichrome staining. (**J**) Representative immunoblots and quantification of the relative protein levels of MR/NR3C2 in DSS rat kidneys. (**K**) Representative immunoblots and quantification of the relative protein levels of renal injury markers (TGF-β1 and KIM-1) in DSS rat kidneys. (**L**) Representative immunoblots and quantification of the relative protein levels of EMT markers (fibronectin, E-cadherin, vimentin, and α-SMA) in DSS rat kidneys. (**M**) Representative immunoblots and quantification of the relative protein levels of proinflammatory mediators (IL-6, TNF-α, and ICAM-1) in DSS rat kidneys. Data are expressed as mean ± SD. ^*^ *P* < 0.05, ^**^ *P* < 0.01, ^***^ *P* < 0.001 vs. NS group; ^#^ *P* < 0.05, ^##^ *P* < 0.01, ^###^ *P* < 0.001 vs. HS group

A model of salt-sensitive hypertension and renal injury was established in DSS rats via an 8-week high-salt diet. Concurrently, varying doses of finerenone (5, 10, and 20 mg/kg/day) were evaluated to determine the optimal dose (Fig. 1A). Compared with the NS group, rats in the HS group exhibited a time-dependent and significant elevation in SBP. Finerenone administration effectively mitigated this hypertensive response, with the 10 mg/kg/day dose demonstrating the most pronounced efficacy (*P* < 0.05; Fig. 1B). Consistently, high-salt-fed rats developed significant renal dysfunction, characterized by elevated levels of BUN, SCr, UACR, and 24-h uALB, all of which were significantly attenuated by finerenone treatment (Fig. 1C–F). Additionally, finerenone mitigated high-salt-induced renal hypertrophy without altering overall body weight (Fig. S1A, B). Histopathological examination further corroborated the structural damage induced by high-salt intake. H&E staining revealed severe glomerular atrophy, tubular dilation, and intratubular cast formation in the HS group. Furthermore, Masson’s trichrome and Sirius Red staining demonstrated extensive collagen deposition in both glomerular and tubulointerstitial regions (Fig. 1G–I).

To elucidate the molecular mechanisms underlying this renoprotective effect, we next examined the protein expression of MR and downstream pathological markers. Immunoblot analysis revealed that high-salt loading triggered an aberrant upregulation of MR, which was effectively suppressed by finerenone administration (Fig. 1J). Parallel to this MR upregulation, the expression of renal injury and fibrotic markers was markedly upregulated in the HS group, whereas finerenone treatment significantly reversed these molecular signatures (Fig. 1K). To examine whether these protective effects involved tubular epithelial cells, we found that finerenone rescued the high-salt-induced viability decline in cultured HK-2 cells (Fig. S1C, D). Furthermore, immunoblotting confirmed that finerenone inhibited high-salt-driven EMT and inflammatory responses consistently both in vivo and in vitro (Fig. 1L, M and S1E, F). Collectively, these multiscale findings demonstrate that pharmacological MR blockade effectively mitigates salt-sensitive renal injury by restraining tubular damage, inflammation, and fibrotic remodeling.

**Fig. 2:**
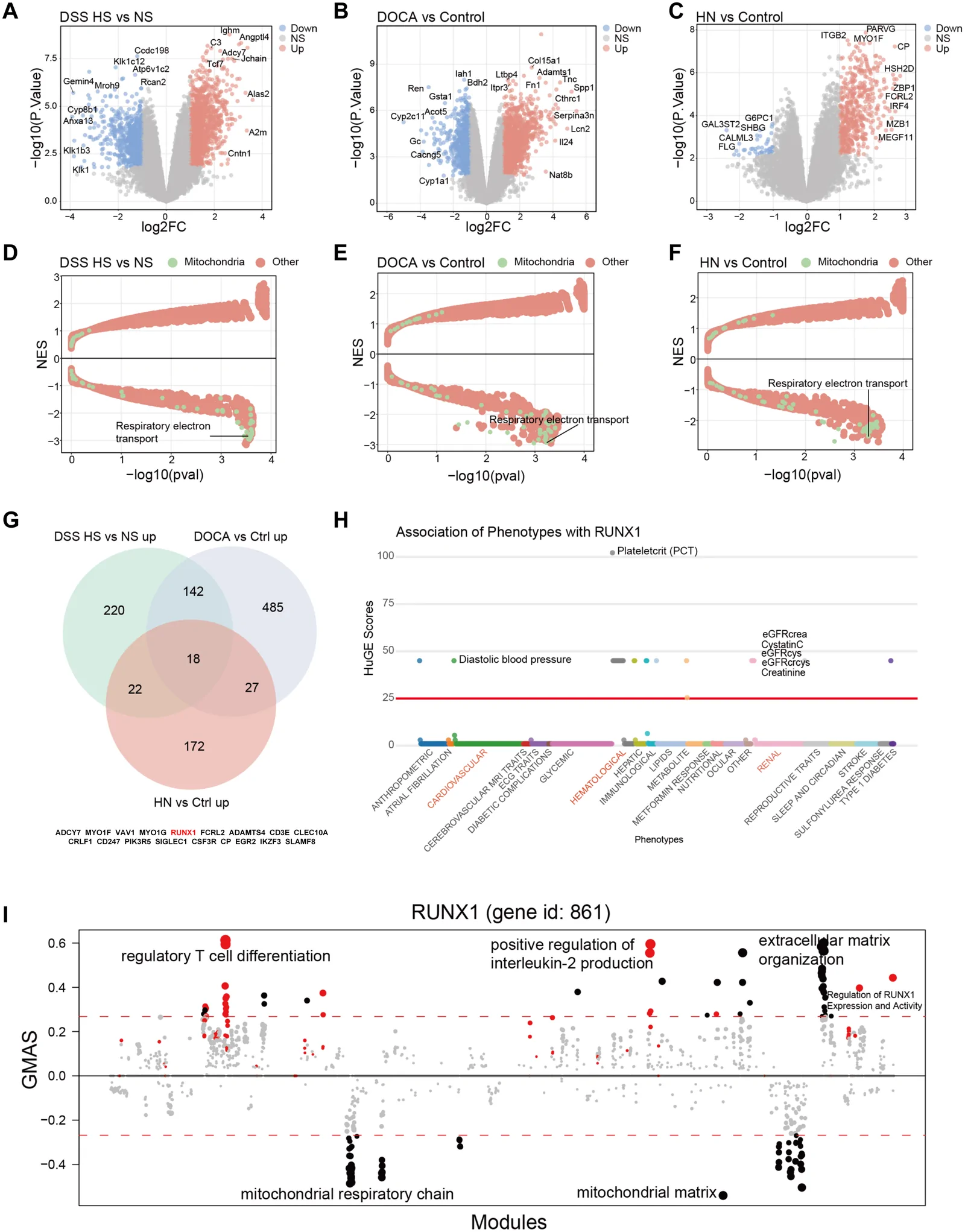
Integrative cross-species transcriptomics links RUNX1 to mitochondrial dysfunction in salt-sensitive hypertensive nephropathy. (**A–C**) Volcano plots showing differentially expressed genes (DEGs) in DSS rats (HS vs. NS; NS group, n = 6; HS group, n = 5), DOCA-salt rats (DOCA vs. Control, n = 4 per group), and human hypertensive nephropathy (HN vs. Control, n = 6 per group). Red and blue dots indicate upregulated and downregulated genes, respectively. (**D–F**) Gene set enrichment analysis (GSEA) for respective comparisons. Green dots highlight mitochondrial pathways, and red dots indicate others. Axes represent −log10 (adjusted P) and normalized enrichment score (NES). (**G**) Intersection of upregulated DEGs across the three comparisons, yielding a conserved 18-gene set. (**H**) HuGeAMP analysis linking RUNX1 to human phenotypes. Scatter plot of HuGE scores colored by category. Horizontal lines mark HuGE score thresholds of 25, 50, 75, and 100. According to HuGeAMP classification, HuGE scores ≥ 25 indicate a ‘very strong’ association. Notably, five renal phenotypes (eGFRcrea, CystatinC, eGFRcys, eGFRcrcys, and Creatinine) and diastolic blood pressure show ‘very strong’ associations (HuGE scores ≥ 25). (**I**) G-MAD analysis of RUNX1, revealing significant negative associations with mitochondrial modules. Dashed lines indicate significance thresholds. Red/black dots represent known/predicted functions.

### Integrative cross-species transcriptomics links RUNX1 to mitochondrial dysfunction in salt-sensitive hypertensive nephropathy

To uncover conserved transcriptomic alterations in hypertensive nephropathy, differential expression analyses were conducted across three independent bulk RNA-seq datasets (Fig. 2A–C): (i) the present DSS rat model, (ii) a public rat dataset of deoxycorticosterone acetate (DOCA)-salt hypertension [34], and (iii) a public human dataset of hypertensive nephropathy [35]. To evaluate the biological significance of these dysregulated genes, gene set enrichment analysis integrating the GO, KEGG, and Reactome databases was performed. Despite species and model differences, all three comparisons consistently revealed significant downregulation of mitochondrial-related pathways in the hypertensive groups versus controls (Fig. 2D–F). By intersecting the upregulated genes across these three datasets (adjusted *P <* 0.05, |log2FC| > 1.5), we identified a conserved 18-gene signature potentially driving disease progression (Fig. 2G). Among these candidates, RUNX1 was prioritized based on its genetic associations with blood pressure and renal function indexes. Specifically, HuGeAMP platform analysis demonstrated that RUNX1 expression was significantly associated with hypertension risk and estimated glomerular filtration rate (eGFR) (Fig. 2H).

To further elucidate the functional role of RUNX1 in the kidney, we performed G-MAD analysis, which integrates 30 renal transcriptomic datasets to assess gene–pathway associations. This analysis revealed significant negative correlations between RUNX1 expression and core mitochondrial modules, including the mitochondrial respiratory chain and mitochondrial matrix (Fig. 2I). Corroborating the G-MAD predictions, correlation profiling across multiple independent kidney transcriptomic cohorts confirmed a consistent inverse association between RUNX1 levels and genes governing the mitochondrial electron transport chain (ETC; Complexes I–IV) and oxidative phosphorylation machinery (Fig. S2).

Collectively, these results nominate RUNX1 as a candidate transcriptional mediator linking salt-sensitive hypertension to renal mitochondrial dysfunction, providing a cross-species bioinformatic foundation for subsequent mechanistic investigation and therapeutic evaluation.

### Finerenone attenuates the MR-RUNX1 transcriptional axis and restores mitochondrial homeostasis under high-salt conditions

**Fig. 3:**
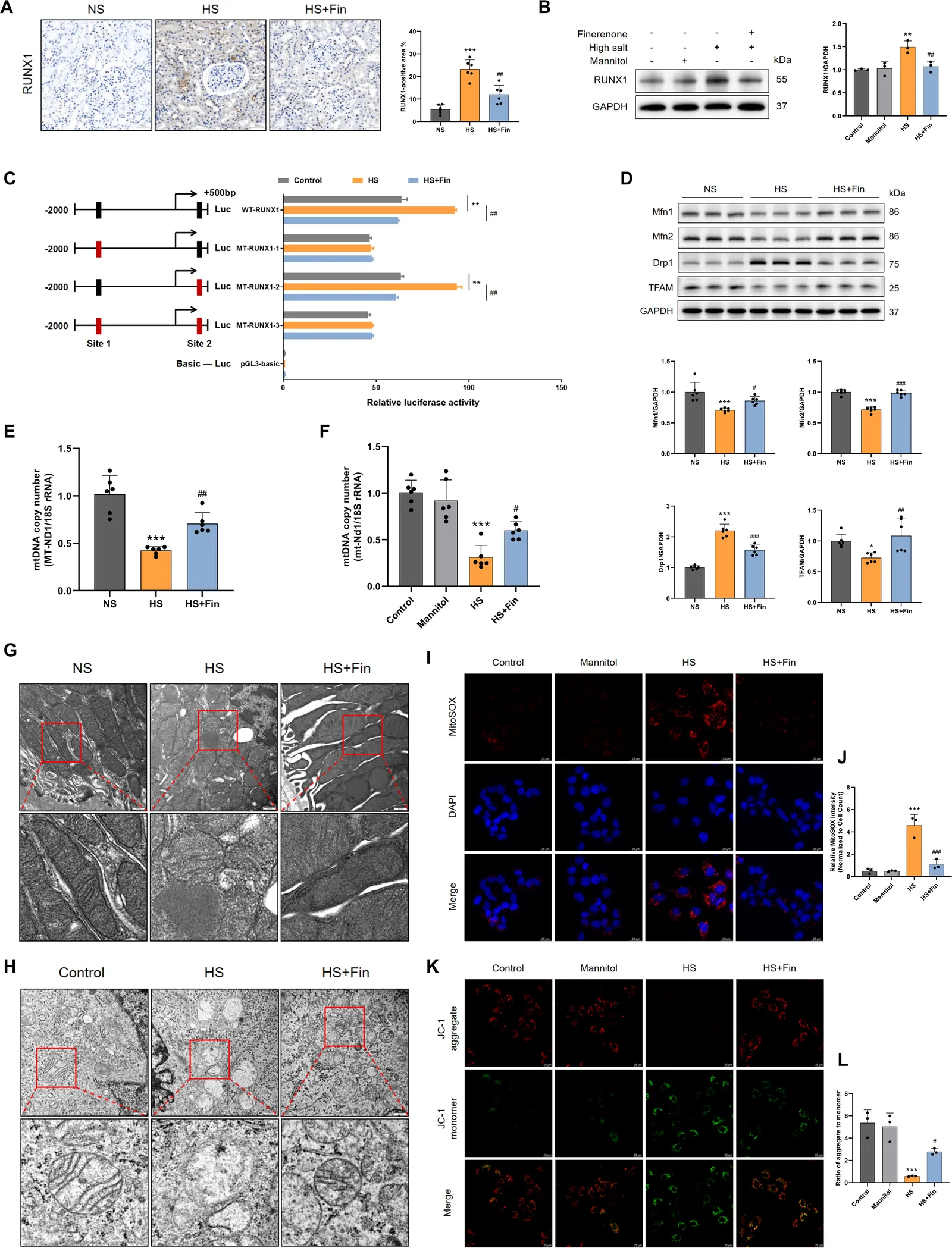
Finerenone attenuates the MR-RUNX1 transcriptional axis and restores mitochondrial homeostasis under high-salt conditions. (A) Representative photomicrographs of immunohistochemical staining for RUNX1 in DSS rat kidneys and quantification of RUNX1-positive areas (*n* = 6 rats per group; scale bar = 50 μm). (**B**) Representative immunoblots and quantification of the relative protein levels of RUNX1 in HK-2 cells. (**C**) Dual-luciferase reporter assays assessing RUNX1 promoter activity. Relative luciferase activity of wild-type or mutant promoters was measured in HK-2 cells under the indicated conditions. (**D**) Representative immunoblots and quantification of the relative protein levels of mitochondrial dynamics and biogenesis markers (MFN1, MFN2, DRP1, and TFAM) in DSS rat kidneys. (**E**) qPCR analysis of relative mtDNA copy number in DSS rat kidneys (*n* = 6 rats per group). (**F**) qPCR analysis of relative mtDNA copy number in HK-2 cells (*n* = 6 independent biological replicates per group). (**G**) Representative TEM images of mitochondrial ultrastructure in DSS rat kidneys (*n* = 3 rats per group; scale bar = 500 nm). (**H**) Representative TEM images of mitochondrial ultrastructure in HK-2 cells (*n* = 3 independent biological replicates; scale bar = 500 nm). (**I**) Representative fluorescence images of MitoSOX Red staining to assess mitoROS production in HK-2 cells (*n* = 3 independent experiments; scale bar = 25 μm). (**J**) Quantification of relative MitoSOX Red fluorescence intensity. (**K**) Representative fluorescence images of JC-1 staining to assess mitochondrial membrane potential (ΔΨm) in HK-2 cells (*n* = 3 independent experiments; scale bar = 25 μm). (**L**) Quantification of the ratio of JC-1 red (aggregates) to green (monomers) fluorescence. Data are expressed as mean ± SD. ^*^ *P* < 0.05, ^**^ *P* < 0.01, ^***^ *P* < 0.001 vs. NS or Control group; ^#^ *P* < 0.05, ^##^ *P* < 0.01, ^###^ *P* < 0.001 vs. HS group

Based on the transcriptomic prioritization of RUNX1, we first characterized its protein expression and histological localization. Compared with NS controls, the HS group exhibited a significant upregulation of RUNX1 in rat kidneys, with its expression predominantly localized to tubular epithelial cells (Fig. 3A). Consistently, immunoblot analysis showed that finerenone significantly attenuated high-salt-induced renal RUNX1 upregulation at 5, 10, and 20 mg/kg/day, with the most pronounced suppression observed at 10 mg/kg/day (Fig. S3A). Parallel to these in vivo findings, high-salt stimulation significantly induced RUNX1 expression in HK-2 cells, whereas mannitol did not recapitulate this effect and finerenone treatment significantly attenuated high-salt-induced RUNX1 upregulation (Fig. 3B).

To further determine whether MR contributes to high-salt-induced RUNX1 promoter activation, dual-luciferase reporter assays were performed. The wild-type RUNX1 promoter exhibited robust basal activity that was significantly enhanced by high-salt stimulation (Fig. 3C). Site-directed mutagenesis of the predicted MR-binding motif at Site 1, but not Site 2, abolished this salt-induced activation, and combined mutation of both sites produced a similar loss of responsiveness. Finerenone treatment effectively suppressed the luciferase activity of the wild-type construct but had no effect on the Site 1 mutant, indicating that this predicted MR-responsive element is required for high-salt-induced RUNX1 promoter activation and its suppression by finerenone.

Having established this MR-dependent RUNX1 promoter response, we next evaluated its impact on mitochondrial homeostasis. Immunoblotting revealed that high-salt exposure shifted mitochondrial dynamics toward excessive fission, characterized by increased dynamin-related protein 1 (DRP1) levels alongside decreased expression of the fusion proteins mitofusin 1 (MFN1) and mitofusin 2 (MFN2), whereas mannitol alone did not induce comparable changes in these mitochondrial dynamics and biogenesis markers (Fig. 3D and S3B). Additionally, the expression of mitochondrial transcription factor A (TFAM)—a master regulator of mitochondrial biogenesis—was significantly downregulated by high salt, leading to a significant reduction of mtDNA copy number in both rat kidneys and HK-2 cells; notably, these effects were rescued by finerenone (Fig. 3E, F). Ultrastructurally, TEM confirmed severe mitochondrial damage in the HS group, presenting as reduced matrix density, swelling, membrane rupture, and cristae vacuolation, all of which were markedly ameliorated by finerenone (Fig. 3G, H). Functionally, finerenone restored mitochondrial bioenergetics, as evidenced by the suppression of mitoROS overproduction and the recovery of mitochondrial membrane potential; notably, mannitol alone did not reproduce these mitochondrial abnormalities (Fig. 3I–L). Collectively, these data indicate that finerenone mitigates high-salt-induced mitochondrial impairment at least in part by suppressing the MR-RUNX1 transcriptional axis.

### RUNX1 contributes to tubular inflammation and EMT under high-salt conditions

**Fig. 4:**
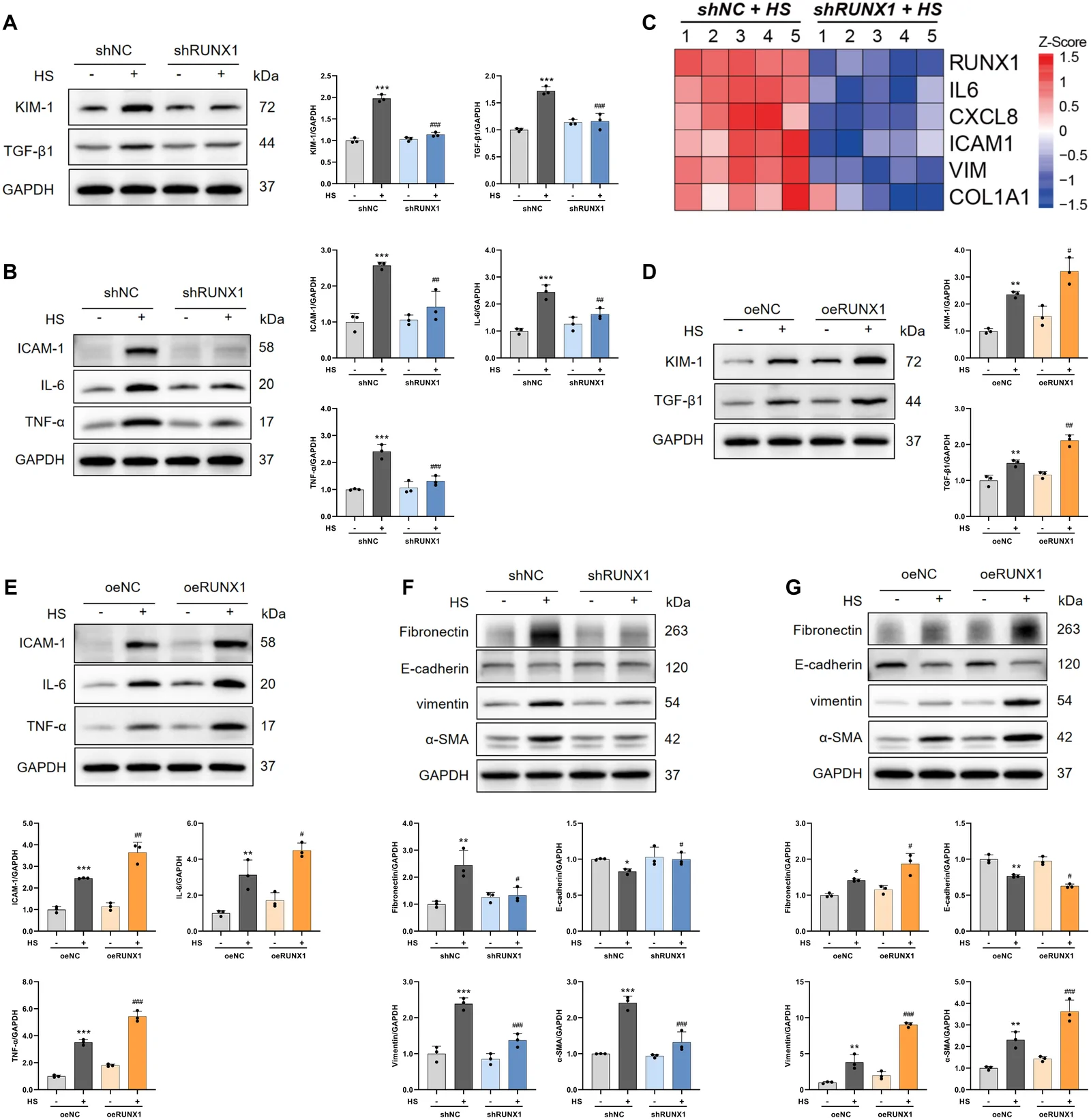
RUNX1 contributes to tubular inflammation and EMT under high-salt conditions. (**A**) Representative immunoblots and quantification of the relative protein levels of renal injury markers (TGF-β1 and KIM-1) in HK-2 cells following RUNX1 knockdown under high-salt (HS) conditions. (**B**) Representative immunoblots and quantification of the relative protein levels of proinflammatory mediators (IL-6, TNF-α, and ICAM-1) in HK-2 cells following RUNX1 knockdown under HS conditions. (**C**) Heatmap visualizing the differential gene expression profile upon RUNX1 knockdown under HS conditions. Data represent Z-score–normalized transcript levels of RUNX1, IL6, CXCL8, ICAM1, VIM, and COL1A1. Each column represents an independent biological replicate (*n* = 6). (**D**) Representative immunoblots and quantification of the relative protein levels of renal injury markers in HK-2 cells following RUNX1 overexpression under HS conditions. (**E**) Representative immunoblots and quantification of the relative protein levels of proinflammatory mediators in HK-2 cells following RUNX1 overexpression under HS conditions. (**F**) Representative immunoblots and quantification of the relative protein levels of EMT markers (fibronectin, E-cadherin, vimentin, and α-SMA) in HK-2 cells following RUNX1 knockdown under HS conditions. (**G**) Representative immunoblots and quantification of the relative protein levels of EMT markers in HK-2 cells following RUNX1 overexpression under HS conditions. Data are expressed as mean ± SD. ^*^ *P* < 0.05, ^**^ *P* < 0.01, ^***^ *P* < 0.001 vs. the shNC or oeNC group; ^#^ *P* < 0.05, ^##^ *P* < 0.01, ^###^ *P* < 0.001 vs. the shNC + HS or oeNC + HS group

We next investigated the functional role of RUNX1 in high-salt-induced tubular injury by performing targeted lentiviral shRNA knockdown in HK-2 cells. Under basal conditions, RUNX1-knockdown (shRUNX1) and control (shNC) cells exhibited comparable levels of kidney injury molecule-1 (KIM-1) and transforming growth factor-β1 (TGF-β1) (Fig. 4A). Following high-salt exposure, however, the marked upregulation of these injury markers and proinflammatory mediators, including interleukin-6 (IL-6), intercellular adhesion molecule-1 (ICAM-1), and tumor necrosis factor-α (TNF-α), observed in shNC cells was significantly attenuated by RUNX1 knockdown (Fig. 4B). Transcriptomic profiling further corroborated these findings, revealing that RUNX1 knockdown reversed inflammation- and injury-associated gene signatures (Fig. 4C). Conversely, RUNX1 overexpression significantly exacerbated the high-salt-induced upregulation of KIM-1 and TGF-β1, as well as the secretion of inflammatory mediators (Fig. 4D, E). Furthermore, RUNX1 knockdown effectively preserved epithelial integrity against high-salt-induced EMT, as evidenced by the restoration of E-cadherin and the concurrent suppression of mesenchymal markers, including vimentin, alpha-smooth muscle actin (α-SMA), and fibronectin (Fig. 4F). In contrast, RUNX1 overexpression significantly potentiated these mesenchymal phenotypic changes (Fig. 4G). Collectively, these data indicate that RUNX1 is involved in high-salt-induced tubular inflammation and EMT.

### RUNX1 contributes to mitochondrial dysfunction and cytosolic mtDNA release under high-salt conditions

**Fig. 5:**
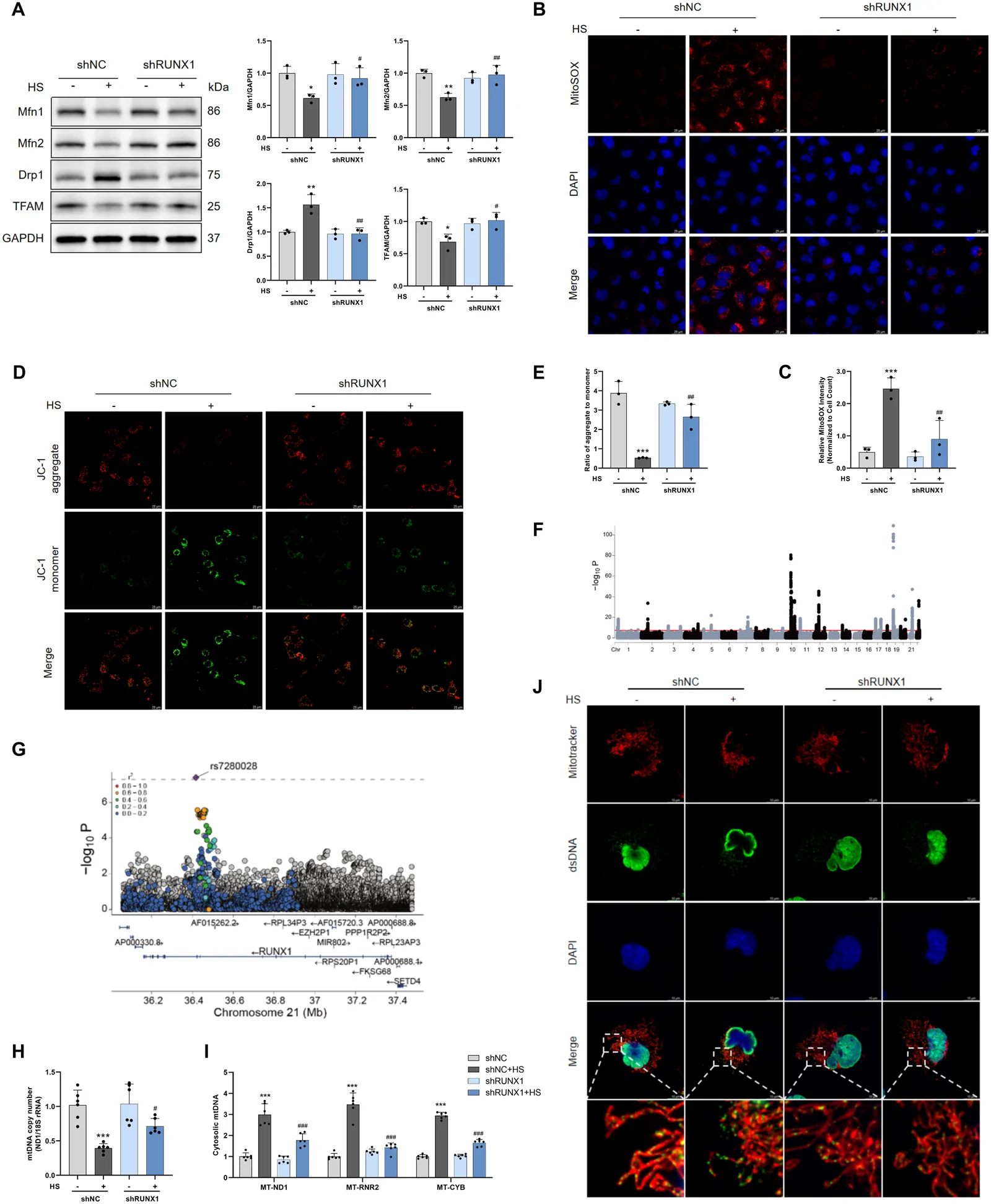
RUNX1 contributes to mitochondrial dysfunction and cytosolic mtDNA release under high-salt conditions. (**A**) Representative immunoblots and quantification of the relative protein levels of mitochondrial dynamics and biogenesis markers (MFN1, MFN2, DRP1, and TFAM) in HK-2 cells following RUNX1 knockdown under high-salt (HS) conditions. (**B**) Representative fluorescence images of MitoSOX Red staining to assess mitoROS production in HK-2 cells following RUNX1 knockdown under HS conditions (*n* = 3 independent experiments; scale bar = 25 μm). (**C**) Quantification of relative MitoSOX Red fluorescence intensity. (**D**) Representative fluorescence images of JC-1 staining to assess mitochondrial membrane potential (ΔΨm) in HK-2 cells following RUNX1 knockdown under HS conditions (*n* = 3 independent experiments; scale bar = 25 μm). (**E**) Quantification of the ratio of JC-1 red (aggregates) to green (monomers) fluorescence. (**F**) Manhattan plot derived from the GWAS analysis of blood mtDNA copy number. Each dot represents a genetic variant; the x-axis displays chromosomal positions (chromosome 1–22), and the y-axis indicates the statistical significance (− log10 *P*-value). The red dashed line denotes the genome-wide significance threshold (*P* = 5 × 10^− 8^). (**G**) Regional association plot for the RUNX1 locus, centered on the lead variant rs7280028. Data points are colored based on linkage disequilibrium (LD, r²) with the lead SNP. Gene annotations are displayed at the bottom. (**H**) qPCR analysis of relative mtDNA copy number in HK-2 cells following RUNX1 knockdown under HS conditions (*n* = 6 independent biological replicates per group). (**I**) qPCR analysis of specific mtDNA fragments (MT-ND1, MT-RNR2, and MT-CYB) in validated cytosolic fractions of HK-2 cells following RUNX1 knockdown under HS conditions (*n* = 6 independent biological replicates per group). (**J**) Representative confocal microscopy images of HK-2 cells co-stained for mitochondria (MitoTracker Red), mtDNA (anti-dsDNA, green), and nuclei (DAPI, blue) to visualize cytosolic mtDNA release (*n* = 3 independent experiments; scale bar = 10 μm). Data are expressed as mean ± SD. ^*^ *P* < 0.05, ^**^ *P* < 0.01, ^***^ *P* < 0.001 vs. the shNC group; ^#^ *P* < 0.05, ^##^ *P* < 0.01, ^###^ *P* < 0.001 vs. the shNC + HS group

To further delineate how RUNX1 affects mitochondrial homeostasis, we assessed genetically modified HK-2 cells. Notably, targeted RUNX1 knockdown effectively reversed the high-salt-induced imbalance in mitochondrial dynamics, as evidenced by the recovery of fission/fusion markers and TFAM expression. Conversely, RUNX1 overexpression further aggravated this fission-dominant phenotype and TFAM decrease (Fig. 5A and S4A). Functionally, RUNX1 deficiency attenuated high-salt-induced mitoROS accumulation and membrane potential depolarization, while its overexpression intensified these pathological alterations (Fig. 5B–E and S4B–E).

Next, our analysis of human GWAS data revealed a significant genome-wide association between the RUNX1 locus and blood mtDNA copy number, with rs7280028 identified as the lead variant (Fig. 5F, G). Consistent with this human genetic evidence, in vitro RUNX1 knockdown significantly reversed the high-salt-induced reduction in mtDNA copy number, whereas its overexpression further exacerbated this loss (Fig. 5H and S4F). Notably, severe mitochondrial stress often culminates in the cytosolic release of mtDNA, where it acts as a potent DAMP to trigger inflammation. To directly quantify this phenomenon, specific mtDNA fragments (MT-ND1, MT-RNR2, and MT-CYB) were assessed in cytosolic fractions validated by fraction purity controls to exclude detectable nuclear and mitochondrial contamination (Fig. S7). High-salt exposure triggered a significant accumulation of cytosolic mtDNA, an effect significantly suppressed by RUNX1 knockdown but amplified by its overexpression (Fig. 5I and S4G). Parallel osmotic-control experiments further showed that mannitol alone did not induce cytosolic accumulation of MT-ND1, MT-RNR2, or MT-CYB (Fig. S3C). Immunofluorescence staining visually corroborated these biochemical findings: the presence of extramitochondrial dsDNA in the cytosol was markedly attenuated by RUNX1 knockdown and intensified by its overexpression (Fig. 5J and S4H). Collectively, these findings support an important role of RUNX1 in regulating mitochondrial integrity and cytosolic mtDNA release under high-salt conditions.

### RUNX1-mediated cytosolic mtDNA release engages the cGAS-STING innate immune pathway

**Fig. 6:**
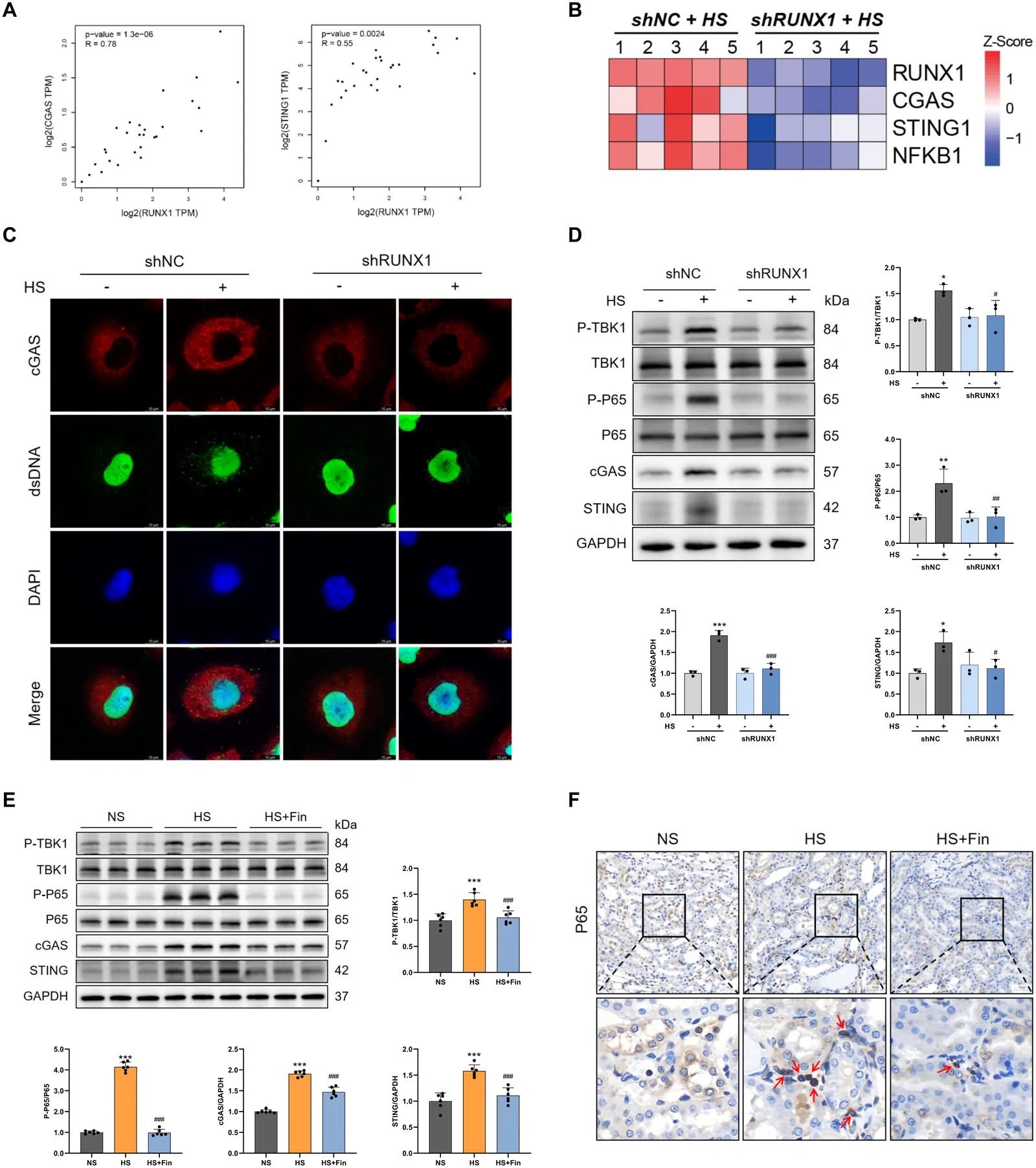
RUNX1-mediated cytosolic mtDNA release engages the cGAS-STING innate immune pathway. (**A**) Scatter plots illustrating the positive correlation between RUNX1 mRNA expression and innate immune sensors (CGAS and STING1). Data are presented as log2-transformed expression levels. (**B**) Heatmap visualization of differential gene expression profiles following RUNX1 knockdown under high-salt (HS) conditions. Z-score–normalized transcript levels of RUNX1, CGAS, STING1, and NFKB1 are displayed for HS-treated control (shNC + HS) and RUNX1-knockdown (shRUNX1 + HS) cells. Each column represents an independent biological replicate (*n* = 6). (**C**) Representative confocal microscopy images displaying the cytosolic colocalization of cGAS (anti-cGAS, red) and mtDNA (anti-dsDNA, green) in HK-2 cells. Nuclei were counterstained with DAPI (blue) (*n* = 3 independent experiments; scale bar = 10 μm). (**D**) Representative immunoblots and quantification of the relative protein levels of the cGAS-STING signaling cascade components (cGAS, STING, p-TBK1/TBK1, and p-p65/p65) in HK-2 cells following RUNX1 knockdown under HS conditions. (**E**) Representative immunoblots and quantification of the relative protein levels of the cGAS-STING signaling cascade components (cGAS, STING, p-TBK1/TBK1, and p-p65/p65) in DSS rat kidneys. (**F**) Representative immunohistochemical images of the nuclear translocation of NF-κB p65 (indicated by arrows) in DSS rat kidneys (*n* = 6 rats per group; scale bar = 10 μm). Data are expressed as mean ± SD. ^*^ *P* < 0.05, ^**^ *P* < 0.01, ^***^ *P* < 0.001 vs. shNC or NS groups; ^#^ *P* < 0.05, ^##^ *P* < 0.01, ^###^ *P* < 0.001 vs. shNC + HS or HS groups

Given that cytosolic mtDNA canonically activates the cGAS-STING innate immune pathway [36], we hypothesized that RUNX1-associated mtDNA release engages this axis and contributes to downstream inflammatory activation. Supporting this hypothesis, analysis of the Genotype-Tissue Expression (GTEx) kidney dataset via Gene Expression Profiling Interactive Analysis (GEPIA) revealed strong positive correlations between RUNX1 expression and the transcript levels of CGAS (*R* = 0.78, *P* = 1.3 × 10⁻⁶) and STING1 (*R* = 0.55, *P* = 0.0024) (Fig. 6A). Consistently, our RNA-seq analysis indicated that high-salt stress significantly upregulated CGAS, STING1, and NFKB1 transcripts in HK-2 cells, an induction effectively blunted by RUNX1 knockdown (Fig. 6B).

To determine whether these transcriptomic alterations translate into functional pathway activation, we assessed the spatial association between cytosolic dsDNA and cGAS. Immunofluorescence assays showed that high-salt exposure promoted the cytosolic colocalization of dsDNA with cGAS, indicating the active sensing of leaked mtDNA. This spatial association was significantly diminished by RUNX1 knockdown but intensified by its overexpression (Fig. 6C and S5A). Downstream immunoblot profiling further supported activation of this signaling cascade, as evidenced by elevated cGAS and STING protein levels, alongside increased phosphorylation of TANK-binding kinase 1 (TBK1) and the NF-κB subunit p65. Consistently, the activation of this pro-inflammatory axis was modulated by RUNX1 expression levels (Fig. 6D and S5B). Parallel osmotic-control experiments further showed that mannitol alone did not activate the cGAS-STING signaling cascade (Fig. S3D).

For the in vivo assessment of this MR-RUNX1-cGAS-STING axis, we evaluated renal tissues from the DSS rat model. Relative to NS controls, high-salt loading provoked a significant upregulation of cGAS and STING proteins, alongside enhanced phosphorylation of TBK1 and p65 (Fig. 6E). Importantly, pharmacological blockade of MR with finerenone effectively suppressed this signaling cascade. Furthermore, immunohistochemical analysis revealed that the high-salt-driven nuclear translocation of p65 was markedly prevented by finerenone treatment (Fig. 6F).

### Pharmacological RUNX1 inhibition mitigates salt-sensitive hypertension and renal injury

**Fig. 7:**
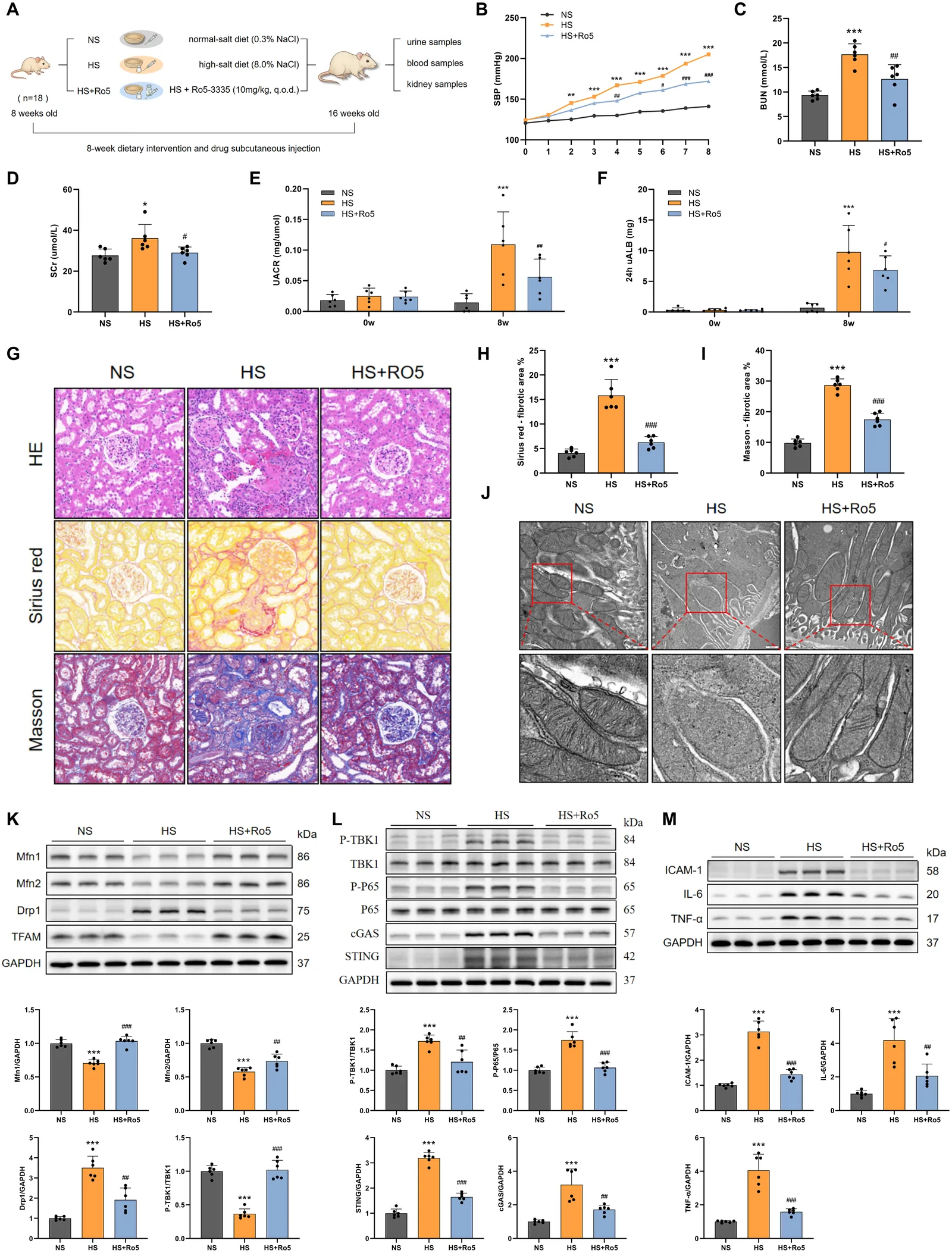
Pharmacological RUNX1 inhibition mitigates salt-sensitive hypertension and renal injury. (**A**) Schematic illustration of the experimental design. DSS rats were maintained on a NS or HS diet for 8 weeks with concomitant vehicle or Ro5-3335 administration via subcutaneous injection (10 mg/kg, every other day). (**B**) Longitudinal monitoring of SBP changes throughout the 8-week intervention period. (**C**) Quantification of BUN levels. (**D**) Quantification of SCr levels. (**E**) Measurement of UACR. (**F**) Measurement of 24-hour uALB excretion. (**G**) Representative photomicrographs of DSS rat kidney sections stained with H&E, Sirius Red, and Masson’s trichrome (*n* = 6 rats per group; scale bar = 50 μm). (**H**) Quantitative analysis of the tubulointerstitial fibrotic area assessed by Sirius Red staining. (**I**) Quantitative analysis of the collagen deposition area assessed by Masson’s trichrome staining. (**J**) Representative TEM images of mitochondrial ultrastructure in DSS rat kidneys (*n* = 3 rats per group; scale bar = 500 nm). (**K**) Representative immunoblots and quantification of the relative protein levels of mitochondrial dynamics and biogenesis markers (MFN1, MFN2, DRP1, and TFAM) in DSS rat kidneys. (**L**) Representative immunoblots and quantification of the relative protein levels of the cGAS-STING signaling cascade components (cGAS, STING, p-TBK1/TBK1, and p-p65/p65) in DSS rat kidneys. (**M**) Representative immunoblots and quantification of the relative protein levels of proinflammatory mediators (IL-6, TNF-α, and ICAM-1) in DSS rat kidneys. Data are expressed as mean ± SD. ^*^ *P* < 0.05, ^**^ *P* < 0.01, ^***^ *P* < 0.001 vs. NS group; ^#^ *P* < 0.05, ^##^ *P* < 0.01, ^###^ *P* < 0.001 vs. HS group

To further assess the translational potential of targeting RUNX1, we evaluated the therapeutic efficacy of Ro5-3335, a small-molecule inhibitor of RUNX1, in the DSS rat model of salt-sensitive hypertension (Fig. 7A). The high-salt vehicle (HS) group developed a severe blood pressure increase, whereas Ro5-3335 treatment (HS+Ro5) partially attenuated the progressive elevation in SBP (Fig. 7B). Consistent with the macroscopic renal hypertrophy induced by high-salt loading, the kidney index was significantly elevated in the HS group but was effectively reduced by Ro5-3335 intervention (Fig. S6A, B). Furthermore, the functional impairments induced by high salt, evidenced by elevated BUN, SCr, UACR, and uALB, were significantly reversed in the HS+Ro5 group (Fig. 7C–F). Histopathological evaluations via H&E, Masson’s trichrome, and Sirius Red staining corroborated these functional benefits, revealing that pharmacological inhibition of RUNX1 significantly mitigated high-salt-induced glomerular and tubular damage, and tubulointerstitial collagen deposition (Fig. 7G–I). Additional endpoint serum biochemical parameters, including ALT, AST, ALB, TP, and LDH, showed no significant differences among the NS, HS, and HS+Ro5 groups (Table S3).

Given our in vitro findings implicating RUNX1 in mitochondrial homeostasis, we next investigated the ultrastructural and molecular alterations of mitochondria in rat kidneys. TEM demonstrated that the severe mitochondrial fragmentation, swelling, and cristae vacuolation observed in the HS group were largely prevented by Ro5-3335 treatment (Fig. 7J). Immunoblotting revealed that Ro5-3335 effectively corrected the high-salt-induced imbalance in mitochondrial dynamics, characterized by the significant downregulation of the pro-fission protein DRP1 and the concomitant restoration of fusion proteins and TFAM (Fig. 7K). Consistently, qPCR analysis of renal tissues confirmed that pharmacological blockade of RUNX1 rescued the significant reduction of total mtDNA copy number induced by high-salt stress, supporting an important role of RUNX1 in mitochondrial genomic instability (Fig. S6C).

We subsequently examined whether the preservation of mitochondrial integrity by Ro5-3335 translated into the suppression of the cGAS-STING innate immune cascade. Immunoblot analysis demonstrated that the high-salt-induced upregulation of cGAS and STING, along with the enhanced phosphorylation of downstream effectors TBK1 and NF-κB p65, were significantly blunted by Ro5-3335 treatment (Fig. 7L). Immunohistochemical analysis revealed that the high-salt-driven nuclear translocation of p65 was markedly prevented by Ro5-3335 administration (Fig. S6D). Consequently, Ro5-3335 significantly suppressed the high-salt-induced upregulation of proinflammatory mediators and renal injury markers (Fig. 7M and S6E). Furthermore, pharmacological inhibition of RUNX1 attenuated the high-salt-induced EMT process, as evidenced by the restoration of E-cadherin and the downregulation of mesenchymal markers (Fig. S6F). Notably, while Ro5-3335 suppressed these downstream pathogenic cascades, it did not alter the upstream high-salt-induced upregulation of MR or the total protein levels of RUNX1 (Fig. S6G, H), consistent with its mechanism as a functional protein-protein interaction inhibitor rather than a transcriptional repressor.

**Fig. 8:**
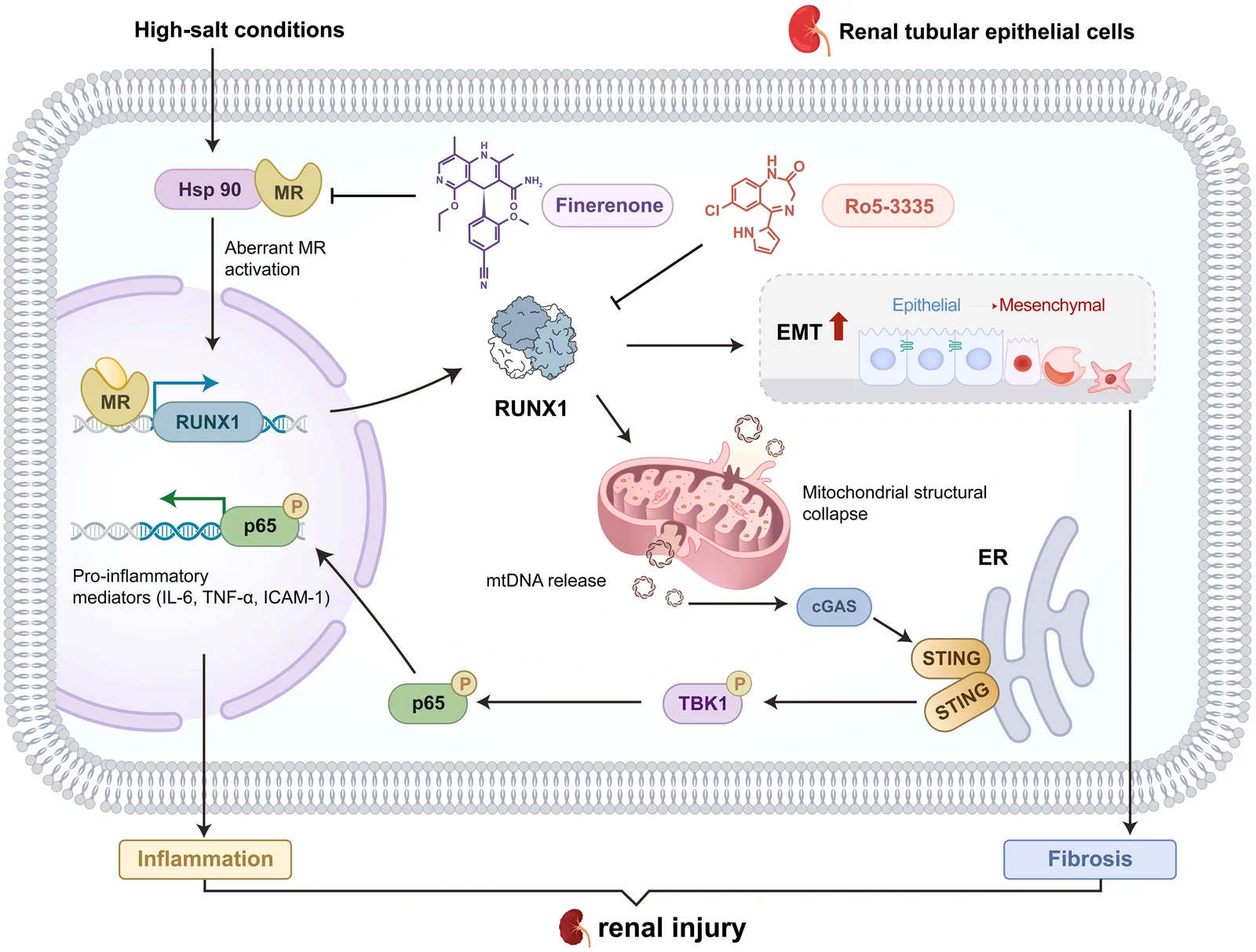
Schematic illustration of the MR-RUNX1 pathogenic axis in salt-sensitive renal injury. Under high-salt conditions, aberrant MR signaling promotes RUNX1 transcriptional upregulation in renal tubular epithelial cells. Sustained RUNX1 induction contributes to mitochondrial homeostatic disruption and cytosolic mtDNA release. The accumulated cytosolic mtDNA acts as a danger signal to engage the cGAS-STING pathway, leading to the phosphorylation of downstream effectors TBK1 and NF-κB p65 and the induction of pro-inflammatory mediators. Concurrently, RUNX1 upregulation promotes EMT. Together, these convergent inflammatory and fibrotic pathways contribute to salt-sensitive renal injury. The MR antagonist finerenone or the RUNX1 inhibitor Ro5-3335 confers renoprotection by intercepting distinct nodes within this pathogenic cascade

Collectively, these integrated in vitro and in vivo findings suggest that the MR-RUNX1-mtDNA-cGAS-STING cascade contributes to high-salt-induced renal injury, providing a mechanistic rationale for the protective effects of finerenone and targeted RUNX1 inhibition (Fig. 8).

## Discussion

The MR is a well-established driver of salt-sensitive hypertension and renal injury; however, the transcriptional mechanisms coupling MR overactivation to intracellular organelle dysfunction and sterile inflammation remain poorly defined. In the present study, integration of human GWAS insights with cross-species transcriptomic profiling points to RUNX1 as an important mediator linking MR signaling to mitochondrial dysfunction. These findings outline a pathogenic model in which the MR-RUNX1 axis contributes to mitochondrial damage and cytosolic mtDNA release, thereby engaging the cGAS-STING surveillance machinery and promoting renal tubular inflammation and fibrotic remodeling. Importantly, we show that pharmacological targeting of this cascade, either via upstream MR antagonism with finerenone or downstream RUNX1 inhibition with Ro5-3335, substantially attenuates mitochondrial dysfunction and renal injury, highlighting the MR-RUNX1 axis as a promising therapeutic target for salt-sensitive renal injury.

The translational relevance of the MR-RUNX1 axis is reinforced by its convergence across cross-species kidney transcriptomics, human genetic resources, and therapeutic intervention. RUNX1 was prioritized in both rodent salt-associated hypertension models and a public human hypertensive nephropathy dataset, with additional human resources linking RUNX1 to mitochondrial and cardiorenal traits. The clinical translational value of dissecting this MR-RUNX1 axis is further consistent with the renal benefits of nonsteroidal MR antagonists. Large-scale clinical trials, such as the FIDELIO-DKD and FIDELITY studies, have demonstrated that finerenone significantly reduces the risk of composite renal endpoints in patients with diabetic kidney disease [37, 38]. More recently, the FIND-CKD trial extended this evidence to adults with CKD without diabetes, showing that finerenone led to a slower decline in eGFR than placebo over 32 months [39]. However, the precise intracellular mechanisms underlying its renoprotective effects—especially under conditions of salt-induced, ligand-independent MR overactivation—have remained incompletely defined. Our preclinical data provide a potential molecular explanation: finerenone may exert part of its organ-protective effects through suppression of MR-dependent RUNX1 upregulation. By elucidating this distinct effector pathway, our study provides a mechanistic rationale for the clinical relevance of finerenone and highlights RUNX1 as a promising downstream target for precision intervention.

Given that our first in vivo cohort compared three finerenone doses, we further considered whether RUNX1 suppression followed a simple dose-dependent pattern. Finerenone significantly attenuated high-salt-induced renal RUNX1 upregulation at all tested doses; however, this effect was not monotonic, as 10 mg/kg/day produced the most pronounced suppression whereas 20 mg/kg/day did not further enhance RUNX1 inhibition. This observation may be interpreted in light of previous finerenone pharmacology studies. Finerenone has been reported to exhibit generally linear pharmacokinetics within the therapeutic dose range [40], whereas population pharmacokinetic and exposure-response modeling suggests that certain downstream pharmacodynamic effects, such as albuminuria reduction, may approach saturation at higher exposures [41]. In addition, the ARTS-DN dose-ranging trial provides clinical background showing that multiple finerenone doses improved UACR in patients with diabetic nephropathy [42]. Therefore, the non-linear RUNX1 response observed here may reflect a downstream pharmacodynamic plateau after sufficient MR blockade, tissue-specific responsiveness of the MR-RUNX1 axis, or compensatory signaling during prolonged high-salt stress. Because we did not measure plasma or renal tissue finerenone concentrations, MR occupancy, or formal pharmacokinetic/pharmacodynamic parameters, this interpretation should remain cautious and warrants future dose-optimization studies.

While RUNX1 is classically recognized as a master regulator of hematopoiesis, its pathological roles in non-immune cells and renal injury have increasingly been recognized [15, 43]. Several lines of evidence support a close relationship between RUNX1 activation, AKI, and renal inflammation. Previous studies have shown that inhibition of RUNX1 expression by endothelial progenitor cell-derived exosomal miR-21-5p alleviates sepsis-induced AKI [17]. In addition, RUNX1 has been identified as a mediator of experimental AKI, in which inflammatory stimuli such as tumor necrosis factor-like weak inducer of apoptosis (TWEAK) and lipopolysaccharide (LPS) induce RUNX1 expression in tubular cells and RUNX1 contributes to inflammatory gene activation [20]. Mechanistically, RUNX1 can interact with NF-κB p50 and amplify Toll-like receptor 4 (TLR4)-triggered inflammatory responses, and it also participates in TGF-β/SMAD3-associated tubular phenotypic transition and fibrotic remodeling [18, 19]. These findings suggest that RUNX1 is not merely a downstream transcriptional target, but may also function as an inflammatory and profibrotic amplifier in injured kidneys.

Aligning with this expanding view of RUNX1 as a renal pathogenic factor, our study independently spotlighted this transcription factor through an unbiased bioinformatics approach. By systematically analyzing cross-species transcriptomic datasets, we identified robust RUNX1 upregulation in salt-sensitive renal injury. Building upon this discovery, we further elucidated a specific upstream regulatory mechanism. Distinct from the classic hemodynamic or ion-transport effectors of MR, such as serum/glucocorticoid-regulated kinase 1 (SGK1), our dual-luciferase reporter assays showed that a predicted MR-responsive element within the RUNX1 promoter is required for high-salt-induced promoter activation and for its suppression by finerenone. This finding places RUNX1 within an MR-responsive transcriptional program in salt-sensitive renal injury.

However, the mechanism of RUNX1 upregulation in renal injury may extend beyond a single upstream transcriptional event. In salt-sensitive renal injury, high salt exposure has been shown to induce persistent inflammatory activation [22], while mitochondrial damage and cytosolic mtDNA release can activate cGAS-STING/NF-κB signaling and promote sterile inflammation [13, 33, 36]. Based on these findings and our own data, we propose a mechanistic model in which aberrant MR activation initially primes RUNX1 induction under high-salt stress, whereas subsequent RUNX1-mediated mitochondrial dysfunction, mtDNA release, and cGAS-STING/NF-κB activation may further reinforce the inflammatory microenvironment. These persistent inflammatory cues, in turn, sustain or amplify RUNX1 expression, forming a feed-forward loop involving MR activation, RUNX1 induction, mitochondrial damage, and renal inflammation. This model may help explain the persistence of tubular injury and fibrotic remodeling under high-salt conditions.

From the perspective of AKI-to-CKD transition, persistent tubular stress, maladaptive repair, unresolved inflammation, mitochondrial dysfunction, and progressive fibrotic remodeling are recognized drivers of chronic renal remodeling after AKI [44]. Although our study was conducted in the setting of salt-sensitive renal injury, the identified MR-RUNX1 pathway overlaps with several of these biological processes. RUNX1 has been implicated in experimental AKI and renal fibrosis, suggesting that sustained RUNX1 activation may connect injury-associated inflammatory signaling with subsequent profibrotic remodeling [18, 20]. Consistently, our data showed that RUNX1 upregulation was associated with mitochondrial dysfunction, cytosolic mtDNA release, cGAS-STING/NF-κB activation, tubular inflammation, and fibrotic remodeling. Previous studies further showed that finerenone protects against ischemia/reperfusion-induced AKI and prevents subsequent chronic deterioration of renal function and structure, partly through reducing oxidative stress [45], while myeloid MR signaling has been implicated in post-injury inflammatory and fibrotic responses [46]. Together, these findings suggest that finerenone-mediated MR blockade may contribute to renoprotection by interrupting the upstream MR-RUNX1 axis and attenuating mitochondrial stress, sterile inflammation, and fibrotic remodeling. Future longitudinal AKI-to-CKD studies will be valuable to directly test this implication.

An important contribution of our study lies in linking RUNX1 to the maintenance of mitochondrial homeostasis, encompassing both structural integrity and genomic stability. Disruption of this balance serves as a key pathogenic driver of renal damage, typically manifesting as reactive oxygen species (ROS) overproduction and impaired mitophagy [47, 48]. Bridging human genetics with cellular pathobiology, GWAS analysis identified a significant association between the RUNX1 locus (rs7280028) and peripheral blood mtDNA copy number, suggesting a fundamental genetic regulation of mitochondrial homeostasis. Intriguingly, while previous studies in acute lung injury suggested that RUNX1 might promote mitophagy to facilitate the clearance of damaged mitochondria [49], our findings in the context of salt-sensitive renal injury reveal a distinctly pathogenic role for this transcription factor. Specifically, sustained RUNX1 upregulation under high-salt stress contributes to deleterious structural and metabolic disruption of the mitochondrial network. This entails not only a shift toward excessive mitochondrial fission, but also a coordinated transcriptional downregulation of nuclear-encoded ETC subunits, leading to marked bioenergetic impairment. Mechanistically, these concurrent defects may overwhelm compensatory mitochondrial quality-control mechanisms. Consequently, the accumulation of uncleared, damaged mitochondria may culminate in the cytosolic release of mtDNA, which acts as a potent DAMP to trigger subsequent innate immune responses.

Central to this immune response is the aberrant engagement of the cGAS-STING surveillance machinery [50]. While evolutionarily conserved to detect microbial pathogens, this sensor system maladaptively recognizes mislocalized endogenous mtDNA as a sterile danger signal under high-salt stress. Upon binding to cytosolic mtDNA, cGAS catalyzes the synthesis of the second messenger cyclic GMP-AMP, which triggers the conformational change and endoplasmic reticulum (ER)-to-Golgi translocation of STING [51, 52]. Consistent with observations in other AKI and CKD models [13, 30], our data provide evidence for this process in salt-sensitive renal injury, showing significantly enhanced cytosolic colocalization of dsDNA with cGAS in renal tubular cells following high-salt challenge. Mechanistically, activated STING serves as a critical signaling adaptor, recruiting TBK1 to initiate a phosphorylation cascade that mobilizes both interferon regulatory factor 3 (IRF3) and NF-κB, thereby establishing a potent proinflammatory feed-forward loop [36, 53]. Consistently, targeted RUNX1 knockdown substantially suppressed this hyperactive axis via upstream interception, effectively diminishing the initial dsDNA-cGAS spatial association. These findings support RUNX1 as an important upstream mediator coupling salt-induced mitochondrial damage to sustained innate immune activation, contributing to the sterile inflammatory phenotype that may promote tubulointerstitial fibrosis.

To further evaluate this RUNX1-associated pathogenic cascade and explore its translational potential, we employed the RUNX1 inhibitor Ro5-3335, which selectively suppresses RUNX1 activity by disrupting its interaction with the co-transcription factor core-binding factor β (CBFβ) [54]. Our in vivo data demonstrated that pharmacological inhibition of RUNX1 effectively mitigated renal damage, preserved mitochondrial integrity, and suppressed the cGAS-STING cascade, despite persistent high-salt-induced MR upregulation. This suggests that RUNX1 represents a functionally relevant node in this pathogenic axis; notably, its pharmacological blockade substantially attenuates downstream inflammatory and fibrotic consequences despite persistent upstream MR overactivation. Supported by recent experimental evidence highlighting the efficacy of Ro5-3335 in mitigating AKI [20], post-myocardial infarction remodeling [21], and pulmonary arterial hypertension [55], our findings support RUNX1 as a therapeutically relevant mediator. Consequently, intercepting this RUNX1-mediated immune cascade provides a mechanistic rationale for mitigating salt-sensitive renal injury.

Nevertheless, several limitations warrant acknowledgement. First, both finerenone and Ro5-3335 reduced SBP in high-salt-fed DSS rats; therefore, in the absence of blood-pressure-matched controls, the relative contributions of blood pressure lowering and direct renal protective mechanisms cannot be fully separated in vivo and should be clarified in future studies incorporating appropriate blood pressure control groups. Second, hematological parameters were not monitored during Ro5-3335 treatment; therefore, potential hematopoietic toxicity associated with prolonged systemic RUNX1 inhibition remains to be specifically evaluated in future studies. Third, our in vivo strategy prioritized pharmacological interventions to approximate clinical therapeutic settings; therefore, future studies using tissue-specific RUNX1 knockout models will be valuable for defining the precise contribution of tubular RUNX1 to renal injury. Fourth, although we focused primarily on tubular epithelial cells, further studies in other renal cell types are required to determine whether the MR-RUNX1 axis also operates in other renal compartments. Finally, while our human genetic and public kidney transcriptomic analyses support the relevance of RUNX1 to mitochondrial and cardiorenal traits, prospective clinical studies are needed to validate the therapeutic implications of the MR-RUNX1 axis in salt-sensitive renal injury.

## Conclusions

In conclusion, our study supports an important role of the MR-RUNX1-mtDNA-cGAS-STING signaling cascade in salt-sensitive renal injury. We show that high-salt-induced MR activation promotes RUNX1 upregulation, which in turn contributes to mitochondrial dysfunction and cytosolic mtDNA release. This subcellular damage engages cGAS-STING-mediated innate immune signaling, accompanied by renal tubular inflammation and fibrotic remodeling. Importantly, both upstream MR antagonism with finerenone and downstream RUNX1 inhibition with Ro5-3335 effectively attenuate this pathological process. Ultimately, these findings provide mechanistic insight into salt-sensitive renal pathogenesis and highlight the therapeutic potential of targeting the MR-RUNX1 axis.

## Supplementary Information

Below is the link to the electronic supplementary material.

Supplementary Material 1

Supplementary Material 2

## Data Availability

The raw rat kidney RNA-sequencing data generated in this study have been deposited in the National Center for Biotechnology Information Sequence Read Archive (SRA) under the BioProject accession number PRJNA1455984. Public transcriptomic datasets used for differential expression analysis were obtained from the Gene Expression Omnibus (GEO), including GSE183841 for DOCA versus control rat kidney samples and GSE166239 for hypertensive nephropathy versus control human kidney samples. Detailed information regarding the public kidney transcriptomic datasets used for differential expression and GeneBridge analyses is summarized in Supplementary Table S4. Additional information regarding the GeneBridge resource is available on the GeneBridge website (https://www.systems-genetics.org/genebridge).

## Abbreviations

AKI: Acute kidney injury
BUN: Blood urea nitrogen
CBFβ: Core-binding factor β
cGAS: Cyclic GMP-AMP synthase
CKD: Chronic kidney disease
DAMP: Damage-associated molecular pattern
DOCA: Deoxycorticosterone acetate
DRP1: Dynamin-related protein 1
DSS: Dahl salt-sensitive
eGFR: estimated glomerular filtration rate
EMT: Epithelial-mesenchymal transition
GEPIA: Gene expression profiling interactive analysis
G-MAD: Gene–module association determination
GTEx: Genotype-tissue expression
GWAS: Genome-wide association study
HK-2: Human renal tubular epithelial cell line
HS: High-salt
ICAM-1: Intercellular adhesion molecule-1
IL-6: Interleukin-6
KIM-1: Kidney injury molecule-1
MFN1: Mitofusin 1
MFN2: Mitofusin 2
mitoROS: mitochondrial reactive oxygen species
MR: Mineralocorticoid receptor
mtDNA: mitochondrial DNA
NF-κB: Nuclear factor kappa B
NS: Normal-salt
RNA-seq: RNA sequencing
RUNX1: Runt-related transcription factor 1
SBP: Systolic blood pressure
SCr: Serum creatinine
STING: Stimulator of interferon genes
TBK1: TANK-binding kinase 1
TEM: Transmission electron microscopy
TFAM: Mitochondrial transcription factor A
TGF-β1: Transforming growth factor-β1
TNF-α: Tumor necrosis factor-α
UACR: Urinary albumin-to-creatinine ratio
uALB: urinary albumin
α-SMA: Alpha-smooth muscle actin

## References

[1] GBD 2019 Risk Factors Collaborators. Global burden of 87 risk factors in 204 countries and territories, 1990–2019: a systematic analysis for the Global Burden of Disease Study 2019. Lancet. 2020;396:1223–49. 10.1016/S0140-6736(20)30752-2 PMC7566194 33069327

[2] NCD Risk Factor Collaboration (NCD-RisC). Worldwide trends in hypertension prevalence and progress in treatment and control from 1990 to 2019: a pooled analysis of 1201 population-representative studies with 104 million participants. Lancet. 2021;398:957–80. 10.1016/S0140-6736(21)01330-1 PMC8446938 34450083

[3] Nishimoto M, Griffin KA, Wynne BM, et al. Salt-Sensitive Hypertension and the Kidney. Hypertension. 2024;81:1206–17. 10.1161/HYPERTENSIONAHA.123.21369 38545804

[4] Bailey MA, Dhaun N. Salt Sensitivity: Causes, Consequences, and Recent Advances. Hypertension. 2024;81:476–89. 10.1161/HYPERTENSIONAHA.123.17959 PMC10863664 37721034

[5] Ayuzawa N, Fujita T. The Mineralocorticoid Receptor in Salt-Sensitive Hypertension and Renal Injury. J Am Soc Nephrol. 2021;32:279–89. 10.1681/ASN.2020071041 PMC8054893 33397690

[6] Hirohama D, Nishimoto M, Ayuzawa N, et al. Activation of Rac1-Mineralocorticoid Receptor Pathway Contributes to Renal Injury in Salt-Loaded db/db Mice. Hypertension. 2021;78:82–93. 10.1161/HYPERTENSIONAHA.121.17263 34058848

[7] González-Juanatey JR, Górriz JL, Ortiz A, et al. Cardiorenal benefits of finerenone: protecting kidney and heart. Ann Med. 2023;55:502–13. 10.1080/07853890.2023.2171110 PMC9891162 36719097

[8] Barrera-Chimal J, Lima-Posada I, Bakris GL, et al. Mineralocorticoid receptor antagonists in diabetic kidney disease - mechanistic and therapeutic effects. Nat Rev Nephrol. 2022;18:56–70. 10.1038/s41581-021-00490-8 34675379

[9] Hoogstraten CA, Hoenderop JG, de Baaij JHF. Mitochondrial Dysfunction in Kidney Tubulopathies. Annu Rev Physiol. 2024;86:379–403. 10.1146/annurev-physiol-042222-025000 38012047

[10] Zhang X, Agborbesong E, Li X. The Role of Mitochondria in Acute Kidney Injury and Chronic Kidney Disease and Its Therapeutic Potential. Int J Mol Sci. 2021;22:11253. 10.3390/ijms222011253 PMC8537003 34681922

[11] Afsar B, Afsar RE. Mitochondrial Damage and Hypertension: Another Dark Side of Sodium Excess. Curr Nutr Rep. 2023;12:495–507. 10.1007/s13668-023-00486-9 37386238

[12] Hasan P, Saotome M, Ikoma T, et al. Mitochondrial fission protein, dynamin-related protein 1, contributes to the promotion of hypertensive cardiac hypertrophy and fibrosis in Dahl-salt sensitive rats. J Mol Cell Cardiol. 2018;121:103–6. 10.1016/j.yjmcc.2018.07.004 29981304

[13] Chung KW, Dhillon P, Huang S, et al. Mitochondrial Damage and Activation of the STING Pathway Lead to Renal Inflammation and Fibrosis. Cell Metab. 2019;30:784–e995. 10.1016/j.cmet.2019.08.003 PMC7054893 31474566

[14] Yao L, Liang X, Liu Y, et al. Non-steroidal mineralocorticoid receptor antagonist finerenone ameliorates mitochondrial dysfunction via PI3K/Akt/eNOS signaling pathway in diabetic tubulopathy. Redox Biol. 2023;68:102946. 10.1016/j.redox.2023.102946 PMC10661120 37924663

[15] Sood R, Kamikubo Y, Liu P. Role of RUNX1 in hematological malignancies. Blood. 2017;129:2070–82. 10.1182/blood-2016-10-687830 PMC5391618 28179279

[16] Lin TC. RUNX1 and cancer. Biochim Biophys Acta Rev Cancer. 2022;1877:188715. 10.1016/j.bbcan.2022.188715 35271994

[17] Zhang Y, Huang H, Liu W, et al. Endothelial progenitor cells-derived exosomal microRNA-21-5p alleviates sepsis-induced acute kidney injury by inhibiting RUNX1 expression. Cell Death Dis. 2021;12:335. 10.1038/s41419-021-03578-y PMC8009943 33785732

[18] Zhou T, Luo M, Cai W, et al. Runt-Related Transcription Factor 1 (RUNX1) Promotes TGF-β-Induced Renal Tubular Epithelial-to-Mesenchymal Transition (EMT) and Renal Fibrosis through the PI3K Subunit p110δ. EBioMedicine. 2018;31:217–25. 10.1016/j.ebiom.2018.04.023 PMC6013935 29759484

[19] Luo MC, Zhou SY, Feng DY, et al. Runt-related Transcription Factor 1 (RUNX1) Binds to p50 in Macrophages and Enhances TLR4-triggered Inflammation and Septic Shock. J Biol Chem. 2016;291:22011–20. 10.1074/jbc.M116.715953 PMC5063984 27573239

[20] Fontecha-Barriuso M, Villar-Gomez N, Guerrero-Mauvecin J, et al. Runt-related transcription factor 1 (RUNX1) is a mediator of acute kidney injury. J Pathol. 2024;264:396–410. 10.1002/path.6355 39472111

[21] Chen H, Wang S, Zhang X, et al. Pharmacological inhibition of RUNX1 reduces infarct size after acute myocardial infarction in rats and underlying mechanism revealed by proteomics implicates repressed cathepsin levels. Funct Integr Genomics. 2024;24:113. 10.1007/s10142-024-01391-2 PMC11166773 38862712

[22] Liao Y, Chu C, Ma Q, et al. Transient high salt intake causes epigenetic changes and leads to persistent inflammatory activation to produce salt memory. J Nutr Biochem. 2023;115:109281. 10.1016/j.jnutbio.2023.109281 36758838

[23] Wang Y, Jia H, Gao K, et al. Renalase alleviates salt-induced kidney necroptosis and inflammation. Hypertens Res. 2024;47:2811–25. 10.1038/s41440-024-01814-4 39117946

[24] Robinson MD, McCarthy DJ, Smyth GK. edgeR: a Bioconductor package for differential expression analysis of digital gene expression data. Bioinformatics. 2010;26:139–40. 10.1093/bioinformatics/btp616 PMC2796818 19910308

[25] Ritchie ME, Phipson B, Wu D, et al. limma powers differential expression analyses for RNA-sequencing and microarray studies. Nucleic Acids Res. 2015;43:e47. 10.1093/nar/gkv007 PMC4402510 25605792

[26] Subramanian A, Tamayo P, Mootha VK, et al. Gene set enrichment analysis: a knowledge-based approach for interpreting genome-wide expression profiles. Proc Natl Acad Sci U S A. 2005;102:15545–50. 10.1073/pnas.0506580102 PMC1239896 16199517

[27] Gupta R, Kanai M, Durham TJ, et al. Nuclear genetic control of mtDNA copy number and heteroplasmy in humans. Nature. 2023;620:839–48. 10.1038/s41586-023-06426-5 PMC10447254 37587338

[28] Costanzo MC, von Grotthuss M, Massung J, et al. The Type 2 Diabetes Knowledge Portal: An open access genetic resource dedicated to type 2 diabetes and related traits. Cell Metab. 2023;35:695–e7106. 10.1016/j.cmet.2023.03.001 PMC10231654 36963395

[29] Li H, Rukina D, David FPA, et al. Identifying gene function and module connections by the integration of multispecies expression compendia. Genome Res. 2019;29:2034–45. 10.1101/gr.251983.119 PMC6886503 31754022

[30] Li J, Sun X, Yang N, et al. Phosphoglycerate mutase 5 initiates inflammation in acute kidney injury by triggering mitochondrial DNA release by dephosphorylating the pro-apoptotic protein Bax. Kidney Int. 2023;103:115–33. 10.1016/j.kint.2022.08.022 36089186

[31] Li M, Yu Y, Xue K, et al. Genistein mitigates senescence of bone marrow mesenchymal stem cells via ERRα-mediated mitochondrial biogenesis and mitophagy in ovariectomized rats. Redox Biol. 2023;61:102649. 10.1016/j.redox.2023.102649 PMC9995482 36871183

[32] Zhang Q, Wei J, Liu Z, et al. STING signaling sensing of DRP1-dependent mtDNA release in kupffer cells contributes to lipopolysaccharide-induced liver injury in mice. Redox Biol. 2022;54:102367. 10.1016/j.redox.2022.102367 PMC9218162 35724543

[33] Maekawa H, Inoue T, Ouchi H, et al. Mitochondrial Damage Causes Inflammation via cGAS-STING Signaling in Acute Kidney Injury. Cell Rep. 2019;29:1261–e736. 10.1016/j.celrep.2019.09.050 31665638

[34] Abedini A, Sánchez-Navaro A, Wu J, et al. Single-cell transcriptomics and chromatin accessibility profiling elucidate the kidney-protective mechanism of mineralocorticoid receptor antagonists. J Clin Invest. 2024;134:e157165. 10.1172/JCI157165 PMC10760974 37906287

[35] Nordbø OP, Landolt L, Eikrem Ø, et al. Transcriptomic analysis reveals partial epithelial-mesenchymal transition and inflammation as common pathogenic mechanisms in hypertensive nephrosclerosis and Type 2 diabetic nephropathy. Physiol Rep. 2023;11:e15825. 10.14814/phy2.15825 PMC10562137 37813528

[36] Hopfner KP, Hornung V. Molecular mechanisms and cellular functions of cGAS-STING signalling. Nat Rev Mol Cell Biol. 2020;21:501–21. 10.1038/s41580-020-0244-x 32424334

[37] Bakris GL, Agarwal R, Anker SD, et al. Effect of finerenone on chronic kidney disease outcomes in type 2 diabetes. N Engl J Med. 2020;383:2219–29. 10.1056/NEJMoa2025845 33264825

[38] Agarwal R, Filippatos G, Pitt B, et al. Cardiovascular and kidney outcomes with finerenone in patients with type 2 diabetes and chronic kidney disease: the FIDELITY pooled analysis. Eur Heart J. 2022;43:474–84. 10.1093/eurheartj/ehab777 PMC8830527 35023547

[39] Heerspink HJL, Neuen BL, Agarwal R, et al. Finerenone in Persons with Chronic Kidney Disease without Diabetes. N Engl J Med. 2026. 10.1056/NEJMoa2604625. 42246672

[40] Heinig R, Eissing T. The pharmacokinetics of the nonsteroidal mineralocorticoid receptor antagonist finerenone. Clin Pharmacokinet. 2023;62:1673–93. 10.1007/s40262-023-01312-9 PMC10684710 37875671

[41] Snelder N, Heinig R, Drenth HJ, et al. Population pharmacokinetic and exposure-response analysis of finerenone: insights based on phase IIb data and simulations to support dose selection for pivotal trials in type 2 diabetes with chronic kidney disease. Clin Pharmacokinet. 2020;59:359–70. 10.1007/s40262-019-00820-x PMC7051931 31583611

[42] Bakris GL, Agarwal R, Chan JC, et al. Effect of finerenone on albuminuria in patients with diabetic nephropathy: a randomized clinical trial. JAMA. 2015;314:884–94. 10.1001/jama.2015.10081 26325557

[43] Riddell A, McBride M, Braun T, et al. RUNX1: an emerging therapeutic target for cardiovascular disease. Cardiovasc Res. 2020;116:1410–23. 10.1093/cvr/cvaa034 PMC7314639 32154891

[44] Yu SMW, Bonventre JV. Acute kidney injury and maladaptive tubular repair leading to renal fibrosis. Curr Opin Nephrol Hypertens. 2020;29:310–18. 10.1097/MNH.0000000000000605 PMC7363449 32205583

[45] Lattenist L, Lechner SM, Messaoudi S, et al. Nonsteroidal mineralocorticoid receptor antagonist finerenone protects against acute kidney injury-mediated chronic kidney disease: role of oxidative stress. Hypertension. 2017;69:870–8. 10.1161/HYPERTENSIONAHA.116.08526 28320854

[46] Barrera-Chimal J, Estrela GR, Lechner SM, et al. The myeloid mineralocorticoid receptor controls inflammatory and fibrotic responses after renal injury via macrophage interleukin-4 receptor signaling. Kidney Int. 2018;93:1344–55. 10.1016/j.kint.2017.12.016 29548765

[47] Li J, Lin Q, Shao X, et al. HIF1α-BNIP3-mediated mitophagy protects against renal fibrosis by decreasing ROS and inhibiting activation of the NLRP3 inflammasome. Cell Death Dis. 2023;14:200. 10.1038/s41419-023-05587-5 PMC10020151 36928344

[48] Fu ZJ, Wang ZY, Xu L, et al. HIF-1α-BNIP3-mediated mitophagy in tubular cells protects against renal ischemia/reperfusion injury. Redox Biol. 2020;36:101671. 10.1016/j.redox.2020.101671 PMC7452120 32829253

[49] Tang X, Zhong L, Tian X, et al. RUNX1 promotes mitophagy and alleviates pulmonary inflammation during acute lung injury. Signal Transduct Target Ther. 2023;8:288. 10.1038/s41392-023-01520-6 PMC10404583 37544975

[50] Dvorkin S, Cambier S, Volkman HE, et al. New frontiers in the cGAS-STING intracellular DNA-sensing pathway. Immunity. 2024;57:718–30. 10.1016/j.immuni.2024.02.019 PMC11013568 38599167

[51] Zhang X, Bai XC, Chen ZJ. Structures and Mechanisms in the cGAS-STING Innate Immunity Pathway. Immunity. 2020;53:43–53. 10.1016/j.immuni.2020.05.013 32668227

[52] Moriyama M, Koshiba T, Ichinohe T, Influenza. A virus M2 protein triggers mitochondrial DNA-mediated antiviral immune responses. Nat Commun. 2019;10:4624. 10.1038/s41467-019-12632-5 PMC6789137 31604929

[53] Fang R, Wang C, Jiang Q, et al. NEMO-IKKβ Are Essential for IRF3 and NF-κB Activation in the cGAS-STING Pathway. J Immunol. 2017;199:3222–33. 10.4049/jimmunol.1700699 28939760

[54] Cunningham L, Finckbeiner S, Hyde RK, et al. Identification of benzodiazepine Ro5-3335 as an inhibitor of CBF leukemia through quantitative high throughput screen against RUNX1-CBFβ interaction. Proc Natl Acad Sci U S A. 2012;109:14592–7. 10.1073/pnas.1200037109 PMC3437880 22912405

[55] Jeong EM, Pereira M, So EY, et al. Targeting RUNX1 as a novel treatment modality for pulmonary arterial hypertension. Cardiovasc Res. 2022;118:3211–24. 10.1093/cvr/cvac001 PMC9799056 35018410

