# Supplementary material for: High salt activates the mineralocorticoid receptor-RUNX1 axis to mediate renal injury via mitochondrial dysfunction
Source: J Transl Med. 2026 Aug 11;24:1410. doi: 10.1186/s12967-026-08805-8 (PMC13746681; doi:10.1186/s12967-026-08805-8)
Supplement: Supplementary file 1 [file 12967_2026_8805_MOESM1_ESM.docx]

**Supplemental Material**

**High salt activates the mineralocorticoid receptor-RUNX1 axis to mediate renal injury via mitochondrial dysfunction**

Table of contents:

- **Figure S1**. Finerenone ameliorates high-salt-induced renal injury.
- **Figure S2**. Conserved negative correlation between RUNX1 and mitochondrial respiratory chain genes across human kidney cohorts.
- **Figure S3**. Finerenone dose-response analysis and mannitol osmotic-control validation of high-salt-induced mitochondrial and inflammatory alterations.
- **Figure S4**. RUNX1 overexpression exacerbates high-salt-induced mitochondrial dysfunction and cytosolic mtDNA release in HK-2 cells.
- **Figure S5**. RUNX1 overexpression amplifies the cGAS-STING innate immune pathway under high-salt conditions.
- **Figure S6**. Pharmacological RUNX1 inhibition mitigates salt-sensitive renal injury.
- **Figure S7.** Quality-control validation of digitonin-based cytosolic fractionation.
- **Table S1**. Details of primary antibodies used for Western blotting and immunohistochemistry.
- **Table S2**. Sequences of primers used for qPCR.
- **Table S3.** Serum biochemical parameters after Ro5-3335 treatment.
- **Table S4**. Public kidney transcriptomic datasets used in this study

**Figure S1. Finerenone ameliorates high-salt-induced renal injury.** (A) Longitudinal body weight changes in DSS rats throughout the 8-week intervention period. (B) Quantification of the kidney index (ratio of kidney weight to body weight) of the DSS rats at the experimental endpoint. (C) Dose-response viability curve of HK-2 cells exposed to increasing concentrations of NaCl (0, 10, 20, 40, 60, and 80 mM) assessed by the Cell Counting Kit-8 (CCK-8) assay. (D) Cell viability assessment of HK-2 cells cotreated with high salt (40 mM NaCl) and indicated concentrations of finerenone (0, 0.2, 0.4, 0.8, 1, 2, 5, and 10 µM). (E) Representative immunoblots and quantification of the relative protein levels of EMT markers (fibronectin, E-cadherin, vimentin, and α-SMA) in HK-2 cells. (F) Representative immunoblots and quantification of the relative protein levels of proinflammatory mediators (IL-6, TNF-α, and ICAM-1) in HK-2 cells. Data are expressed as mean ± SD. ^*^ *P* < 0.05, ^**^ *P* < 0.01, ^***^ *P* < 0.001 vs. the NS or Control group; ^#^ *P* < 0.05, ^##^ *P* < 0.01, ^###^ *P* < 0.001 vs. the HS group.

**Figure S2. Conserved negative correlation between RUNX1 and mitochondrial respiratory chain genes across human kidney cohorts.** Heatmap depicts the correlation between RUNX1 expression and a curated set of mitochondria-related genes across multiple independent kidney transcriptomic cohorts. Each tile represents the RUNX1–gene correlation estimated within a given dataset, red denotes positive correlation and blue denotes negative correlation. Statistical significance is indicated by asterisks: ^*^*P* < 0.05, ^**^*P* < 0.01, and ^***^*P* < 0.001. Rows comprise genes encoding subunits of mitochondrial electron transport chain complexes I–IV as well as additional oxidative phosphorylation (OXPHOS) components, collected from Gene Ontology datasets. Genes and datasets are hierarchically clustered based on correlation profiles to illustrate conserved co-expression patterns across cohorts.

**Figure S3. Finerenone dose-response analysis and mannitol osmotic-control validation of high-salt-induced mitochondrial and inflammatory alterations.** (A) Representative immunoblots and quantification of the relative protein levels of RUNX1 in DSS rat kidneys treated with different doses of finerenone. (B) Representative immunoblots and quantification of the relative protein levels of mitochondrial dynamics and biogenesis markers (MFN1, MFN2, DRP1, and TFAM) in HK-2 cells. (C) qPCR analysis of specific mtDNA fragments (MT-ND1, MT-RNR2, and MT-CYB) in validated cytosolic fractions of HK-2 cells (n = 6 independent biological replicates per group). (D) Representative immunoblots and quantification of the relative protein levels of the cGAS-STING signaling cascade components (cGAS, STING, p-TBK1/TBK1, and p-p65/p65) in HK-2 cells. Data are expressed as mean ± SD. ^*^ *P* < 0.05, ^**^ *P* < 0.01, ^***^ *P* < 0.001 vs. the NS or Control group; ^#^ *P* < 0.05, ^##^ *P* < 0.01, ^###^ *P* < 0.001 vs. the HS group.

**Figure S4. RUNX1 overexpression exacerbates high-salt-induced mitochondrial dysfunction and cytosolic mtDNA release in HK-2 cells.** (A) Representative immunoblots and quantification of the relative protein levels of mitochondrial dynamics and biogenesis markers (MFN1, MFN2, DRP1, and TFAM) in HK-2 cells following RUNX1 overexpression under HS conditions. (B) Representative fluorescence images of MitoSOX Red staining to assess mitoROS production in HK-2 cells following RUNX1 overexpression under HS conditions (n = 3 independent experiments; scale bar = 25 μm). (C) Quantification of relative MitoSOX Red fluorescence intensity. (D) Representative fluorescence images of JC-1 staining to assess mitochondrial membrane potential (ΔΨm) in HK-2 cells following RUNX1 overexpression under HS conditions (n = 3 independent experiments; scale bar = 25 μm). (E) Quantification of the ratio of JC-1 red (aggregates) to green (monomers) fluorescence. (F) qPCR analysis of relative mtDNA copy number in HK-2 cells following RUNX1 overexpression under HS conditions (n = 6 independent biological replicates per group). (G) qPCR analysis of specific mtDNA fragments (MT-ND1, MT-RNR2, and MT-CYB) in validated cytosolic fractions of HK-2 cells following RUNX1 overexpression under HS conditions (n = 6 independent biological replicates per group). (H) Representative confocal microscopy images of HK-2 cells co-stained for mitochondria (MitoTracker Red), mtDNA (anti-dsDNA, green), and nuclei (DAPI, blue) to visualize cytosolic mtDNA release (n = 3 independent experiments; scale bar = 10 μm). Data are expressed as mean ± SD. ^*^ *P* < 0.05, ^**^ *P* < 0.01, ^***^ *P* < 0.001 vs. the oeNC group; ^#^ *P* < 0.05, ^##^ *P* < 0.01, ^###^ *P* < 0.001 vs. the oeNC+HS group.

**Figure S5. RUNX1 overexpression amplifies the cGAS-STING innate immune pathway under high-salt conditions.** (A) Representative confocal microscopy images displaying the cytosolic colocalization of cGAS (anti-cGAS, red) and mtDNA (anti-dsDNA, green) in HK-2 cells. Nuclei were counterstained with DAPI (blue) (n = 3 independent experiments; scale bar = 10 μm). (B) Representative immunoblots and quantification of the relative protein levels of the cGAS-STING signaling cascade components (cGAS, STING, p-TBK1/TBK1, and p-p65/p65) in HK-2 cells following RUNX1 overexpression under HS conditions. Data are expressed as mean ± SD. ^*^ *P* < 0.05, ^***^ *P* < 0.001 vs. the oeNC group; ^#^ *P* < 0.05, ^###^ *P* < 0.001 vs. the oeNC+HS group.

**Figure S6. Pharmacological RUNX1 inhibition mitigates salt-sensitive renal injury.** (A) Longitudinal body weight changes in DSS rats throughout the 8-week intervention period. (B) Quantification of the kidney index (ratio of kidney weight to body weight) of the DSS rats at the experimental endpoint. (C) qPCR analysis of relative mtDNA copy number in DSS rat kidneys (n = 6 rats per group). (D) Representative immunohistochemical images of the nuclear translocation of NF-κB p65 (indicated by arrows) in DSS rat kidneys (n = 6 rats per group; scale bar = 10 μm). (E) Representative immunoblots and quantification of the relative protein levels of renal injury markers (TGF-β1 and KIM-1) in DSS rat kidneys. (F) Representative immunoblots and quantification of the relative protein levels of EMT markers (fibronectin, E-cadherin, vimentin, and α-SMA) in DSS rat kidneys. (G) Representative immunoblots and quantification of the relative protein levels of MR/NR3C2 in DSS rat kidneys. (H) Representative immunoblots and quantification of the relative protein levels of RUNX1 in DSS rat kidneys. Data are expressed as mean ± SD. ^***^ *P* < 0.001 vs. the NS group; ^#^ *P* < 0.05, ^##^ *P* < 0.01, ^###^ *P* < 0.001 vs. the HS group.

**Figure S7. Quality-control validation of digitonin-based cytosolic fractionation.** Representative immunoblots for fraction purity validation in total cell lysates and cytosolic fractions from HK-2 cells under (A) Control, Mannitol, HS, and HS+Fin conditions; (B) shNC and shRUNX1 conditions with or without HS exposure; and (C) oeNC and oeRUNX1 conditions with or without HS exposure. GAPDH, Lamin B1, and COX IV were used as cytosolic, nuclear, and mitochondrial markers, respectively. The absence or minimal detection of Lamin B1 and COX IV in cytosolic fractions supports the purity of the cytosolic fractions and indicates minimal nuclear or mitochondrial contamination during digitonin-based fractionation.

**Table S1.** Details of primary antibodies used for Western blotting and immunohistochemistry.

| Primary antibody | Source; Cat. No.; Dilution |
| --- | --- |
| anti-NR3C2 | Proteintech; 21854-1-AP; 1:1000 |
| anti-RUNX1 | Proteintech; 25315-1-AP; 1:1000 |
| anti-KIM-1 | Proteintech; 30948-1-AP; 1:500 |
| anti-TGF-β1 | Proteintech; 81746-2-RR; 1:5000 |
| anti-Fibronectin | Proteintech; 15613-1-AP; 1:2000 |
| anti-E-cadherin | UpingBio; YP-Ab-17017; 1:1000 |
| anti-Vimentin | Proteintech; 10366-1-AP; 1:20000 |
| anti-α-SMA | Proteintech; 14395-1-AP; 1:2000 |
| anti-ICAM-1 | UpingBio; YP-Ab-16292; 1:1000 |
| anti-IL-6 | Proteintech; 21865-1-AP; 1:1000 |
| anti-TNF-α | Wanleibio; WL01581; 1:1000 |
| anti-MFN1 | Proteintech; 13798-1-AP; 1:2000 |
| anti-MFN2 | Proteintech; 12186-1-AP; 1:5000 |
| anti-DRP1 | Proteintech; 12957-1-AP; 1:2000 |
| anti-TFAM | Abcam; ab307302; 1:1000 |
| anti-cGAS | UpingBio; YP-Ab-17228; 1:1000 |
| anti-STING | Nature Biosciences; A14303; 1:1000 |
| anti-TBK1 | Nature Biosciences; A41951; 1:1000 |
| anti-p-TBK1 | Nature Biosciences; A38779; 1:1000 |
| anti-NF-κB p65 | Nature Biosciences; A66433; 1:1000 |
| anti-p-NF-κB p65 | Nature Biosciences; A19346; 1:1000 |
| anti-GAPDH | Proteintech; 10494-1-AP; 1:5000 |
| anti-Lamin B1 | Nature Biosciences; A72089; 1:1000 |
| anti-COX IV | Proteintech; 11242-1-AP; 1:5000 |

**Table S2**. Sequences of primers used for qPCR.

| Gene name | Species | Direction | Sequence (5’ - 3’) |
| --- | --- | --- | --- |
| GAPDH | Human | Forward | CCCTCAACGACCACTTTGTCA |
|  |  | Reverse | TTCCTCTTGTGCTCTTGCTGG |
| RUNX1 | Human | Forward | TGAGCTGAGAAATGCTACCGC |
|  |  | Reverse | ACTTCGACCGACAAACCTGAG |
| MT-ND1 | Human | Forward | CACTTTCCACACAGACATCA |
|  |  | Reverse | TGGTTAGGCTGGTGTTAGGG |
| MT-CYB | Human | Forward | AACTTCGGCTCACTCCTTGG |
|  |  | Reverse | GATGAAAAGGCGGTTGAGGC |
| MT-RNR2 | Human | Forward | GTTACCCTAGGGATAACAGCGC |
|  |  | Reverse | GATCCAACATCGAGGTCGTAAACC |
| 18S rRNA | Human | Forward | CAGCCACCCGAGATTGAGCA |
|  |  | Reverse | TAGTAGCGACGGGCGGTGTG |
| mt-Nd1 | Rat | Forward | CGTTGCCCAAACCATCTCT |
|  |  | Reverse | CTATTGGTCAGGCGGGGA |
| 18S rRNA | Rat | Forward | TGCGGAAGGATCATTAACGGA |
|  |  | Reverse | AGTAGGAGAGGAGCGAGCGACC |

**Table S3**. Serum biochemical parameters after Ro5-3335 treatment.

| Parameter | NS | HS | HS+Ro5 | *P*-value |
| --- | --- | --- | --- | --- |
| ALT, U/L | 37.52 ± 12.67 | 43.01 ± 10.58 | 38.79 ± 12.22 | 0.708 |
| AST, U/L | 87.46 ± 26.06 | 79.46 ± 15.90 | 82.55 ± 17.66 | 0.793 |
| TP, g/L | 55.61 ± 7.01 | 52.82 ± 6.50 | 55.72 ± 4.74 | 0.661 |
| ALB, g/L | 34.66 ± 2.95 | 33.85 ± 4.81 | 34.32 ± 2.02 | 0.921 |
| LDH, U/L | 310.77 ± 358.65 | 171.49 ± 55.34 | 161.25 ± 52.56 | 0.415 |

Data are expressed as mean ± SD (n = 6 rats per group). Statistical comparisons were performed using one-way ANOVA. Abbreviations: ALT, alanine aminotransferase; AST, aspartate aminotransferase; ALB, albumin; TP, total protein; LDH, lactate dehydrogenase.

**Table S4.** Public kidney transcriptomic datasets used in the present study.

| Source | Species | Identifier | Tissue | Sample size | Analysis purpose |
| --- | --- | --- | --- | --- | --- |
| GEO | Rat | GSE183841 | Kidney | 8 | DEA |
| GEO | Human | GSE166239 | Kidney | 12 | DEA |
| GEO | Human | GSE98320 | Kidney | 1208 | GeneBridge |
| GEO | Human | GSE43974 | Kidney | 554 | GeneBridge |
| GEO | Human | GSE36059 | Kidney | 411 | GeneBridge |
| GEO | Human | GSE48581 | Kidney | 306 | GeneBridge |
| GEO | Human | GSE21374 | Kidney | 282 | GeneBridge |
| GEO | Human | GSE2109 | Kidney | 280 | GeneBridge |
| GEO | Human | GSE76882 | Kidney | 274 | GeneBridge |
| GEO | Human | GSE73731 | Kidney | 265 | GeneBridge |
| GEO | Human | GSE110169 | Kidney | 243 | GeneBridge |
| GEO | Human | GSE31403 | Kidney | 224 | GeneBridge |
| GEO | Human | GSE40435 | Kidney | 202 | GeneBridge |
| GEO | Human | GSE72925 | Kidney | 168 | GeneBridge |
| GEO | Human | GSE17895 | Kidney | 160 | GeneBridge |
| GEO | Human | GSE95611 | Kidney | 154 | GeneBridge |
| GEO | Human | GSE10320 | Kidney | 144 | GeneBridge |
| GEO | Human | GSE53757 | Kidney | 144 | GeneBridge |
| GEO | Human | GSE41137 | Kidney | 140 | GeneBridge |
| GEO | Human | GSE65615 | Kidney | 138 | GeneBridge |
| GEO | Human | GSE46699 | Kidney | 130 | GeneBridge |
| GEO | Human | GSE17818 | Kidney | 115 | GeneBridge |
| GEO | Human | GSE37171 | Kidney | 115 | GeneBridge |
| GEO | Human | GSE50058 | Kidney | 101 | GeneBridge |
| GEO | Human | GSE20491 | Kidney | 99 | GeneBridge |
| GEO | Human | GSE68127 | Kidney | 99 | GeneBridge |
| GEO | Human | GSE47185 | Kidney | 97 | GeneBridge |
| GEO | Human | GSE61739 | Kidney | 96 | GeneBridge |
| GEO | Human | GSE11166 | Kidney | 95 | GeneBridge |
| GEO | Human | GSE15641 | Kidney | 92 | GeneBridge |
| GEO | Human | GSE38262 | Kidney | 92 | GeneBridge |
| GEO | Human | GSE25902 | Kidney | 85 | GeneBridge |
| GEO | Human | GSE32591-Tub | Kidney | 47 | GeneBridge |
| GEO | Human | GSE32591-Glom | Kidney | 46 | GeneBridge |
| GEO | Human | GSE78806 | Kidney | 41 | GeneBridge |

Abbreviations: DEA, differential expression analysis.
